# Reactive astrocytes in ALS display diminished intron retention

**DOI:** 10.1093/nar/gkab115

**Published:** 2021-03-04

**Authors:** Oliver J Ziff, Doaa M Taha, Hamish Crerar, Benjamin E Clarke, Anob M Chakrabarti, Gavin Kelly, Jacob Neeves, Giulia E Tyzack, Nicholas M Luscombe, Rickie Patani

**Affiliations:** The Francis Crick Institute, 1 Midland Road, London, NW1 1AT, UK; Department of Neuromuscular Diseases, Queen Square Institute of Neurology, University College London, London, WC1N 3BG, UK; National Hospital for Neurology and Neurosurgery, University College London NHS Foundation Trust, London, WC1N 3BG, UK; The Francis Crick Institute, 1 Midland Road, London, NW1 1AT, UK; Department of Neuromuscular Diseases, Queen Square Institute of Neurology, University College London, London, WC1N 3BG, UK; Department of Zoology, Faculty of Science, Alexandria University, Alexandria 21511, Egypt; The Francis Crick Institute, 1 Midland Road, London, NW1 1AT, UK; Department of Neuromuscular Diseases, Queen Square Institute of Neurology, University College London, London, WC1N 3BG, UK; The Francis Crick Institute, 1 Midland Road, London, NW1 1AT, UK; Department of Neuromuscular Diseases, Queen Square Institute of Neurology, University College London, London, WC1N 3BG, UK; The Francis Crick Institute, 1 Midland Road, London, NW1 1AT, UK; UCL Genetics Institute, University College London, Gower Street, London WC1E 6BT, UK; The Francis Crick Institute, 1 Midland Road, London, NW1 1AT, UK; The Francis Crick Institute, 1 Midland Road, London, NW1 1AT, UK; Department of Neuromuscular Diseases, Queen Square Institute of Neurology, University College London, London, WC1N 3BG, UK; The Francis Crick Institute, 1 Midland Road, London, NW1 1AT, UK; Department of Neuromuscular Diseases, Queen Square Institute of Neurology, University College London, London, WC1N 3BG, UK; The Francis Crick Institute, 1 Midland Road, London, NW1 1AT, UK; UCL Genetics Institute, University College London, Gower Street, London WC1E 6BT, UK; Okinawa Institute of Science & Technology Graduate University, Okinawa 904-0495, Japan; The Francis Crick Institute, 1 Midland Road, London, NW1 1AT, UK; Department of Neuromuscular Diseases, Queen Square Institute of Neurology, University College London, London, WC1N 3BG, UK; National Hospital for Neurology and Neurosurgery, University College London NHS Foundation Trust, London, WC1N 3BG, UK

## Abstract

Reactive astrocytes are implicated in amyotrophic lateral sclerosis (ALS), although the mechanisms controlling reactive transformation are unknown. We show that decreased intron retention (IR) is common to human-induced pluripotent stem cell (hiPSC)-derived astrocytes carrying ALS-causing mutations in *VCP, SOD1* and *C9orf72*. Notably, transcripts with decreased IR and increased expression are overrepresented in reactivity processes including cell adhesion, stress response and immune activation. This was recapitulated in public-datasets for (i) hiPSC-derived astrocytes stimulated with cytokines to undergo reactive transformation and (ii) *in vivo* astrocytes following selective deletion of TDP-43. We also re-examined public translatome sequencing (TRAP-seq) of astrocytes from a *SOD1* mouse model, which revealed that transcripts upregulated in translation significantly overlap with transcripts exhibiting decreased IR. Using nucleocytoplasmic fractionation of *VCP* mutant astrocytes coupled with mRNA sequencing and proteomics, we identify that decreased IR in nuclear transcripts is associated with enhanced nonsense mediated decay and increased cytoplasmic expression of transcripts and proteins regulating reactive transformation. These findings are consistent with a molecular model for reactive transformation in astrocytes whereby poised nuclear reactivity-related IR transcripts are spliced, undergo nuclear-to-cytoplasmic translocation and translation. Our study therefore provides new insights into the molecular regulation of reactive transformation in astrocytes.

## Graphical Abstract

**Graphical abstract:**
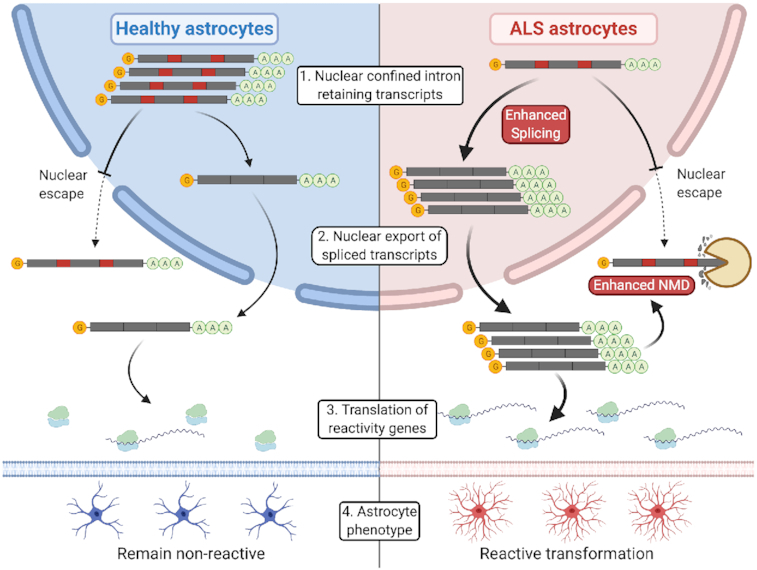
Healthy astrocytes (left, blue) have prevalent nuclear intron retention in reactivity transcripts, promoting their nuclear confinement (introns shown as red rectangles). Conversely, ALS astrocytes (right, red) exhibit decreased intron retention in reactivity transcripts, enabling cytoplasmic translocation and translation upon engaging ribosomes, underlying their reactive transformation. A small number of intron retaining transcripts escape to the cytoplasm where they are degraded by nonsense mediated decay (NMD), which is enhanced in ALS astrocytes.

## INTRODUCTION

Amyotrophic lateral sclerosis (ALS) is a rapidly progressive and incurable neurodegenerative disease. The pathological hallmark of ALS is cytoplasmic aggregation of the RNA-binding protein, transactive response DNA-binding protein 43 (TDP-43, encoded by *TARDBP*) accompanied by loss of nuclear TDP-43, in the motor neurons of >97% of ALS cases (1). Since the identification of the *SOD1* gene mutation in ALS, which accounts for ∼15% of familial cases, ∼30 gene mutations have been discovered of which *C9orf72* is the most common (∼35% of familial cases) (2,3). Valosin-containing protein (*VCP)* is a transitional endoplasmic reticulum ATPase essential for maturation of ubiquitin-containing autophagosomes, and accounts for ∼2% of familial ALS (4). Although motor neuron degeneration was traditionally regarded as the cellular substrate of ALS pathogenesis, accumulating evidence implicates astrocytes, which play vital roles in providing neuronal support including metabolite provision and modulating the immune response (5–7). ALS astrocytes can non-cell autonomously impair motor neuron function through loss of supportive and/or gain of toxic factors, and recent studies additionally revealed cell autonomous astrocyte phenotypes (8–17). ALS postmortem studies consistently report neuroinflammation and reactive gliosis (18–23). Neuroinflammation induces quiescent astrocytes to undergo dramatic changes in gene expression, morphology and function, called ‘reactive transformation’ (24–26). Using inflammatory cues to mimic activated microglia, Liddelow *et al.* profiled astrocyte gene expression changes accompanying reactive transformation and categorized them into pan-reactive, A1 (harmful) and A2 (protective) responses (24,27–28).

Although gene expression changes accompanying reactive responses have been extensively studied, the regulating molecular mechanisms are unknown (24,26–27,29). Alternative splicing, particularly intron retention (IR), is recognized as a major regulator of gene expression during both differentiation and activation of several cell types (30–36). Although other modes of alternative splicing (exon skipping and alternative splice site usage) typically enhance protein synthesis, IR can serve to suppress translation—introns often contain premature termination codons promoting degradation via nonsense-mediated decay (NMD) (36,37). IR can also regulate RNA localization by promoting nuclear confinement, further suppressing translation (31–32,38–40). We recently reported aberrant IR in human-induced pluripotent stem cell (hiPSC)-derived ALS motor neuron cultures undergoing differentiation (41,42). IR has also been implicated in the function of other neuronal and immune cells—orchestrated IR regulates activation of macrophages, granulocytes and lymphocytes—however, its role in astrocytes is unknown (30,33–34,36,42–47).

We sought to determine if alternative splicing, and specifically IR, plays a role in astrocyte reactivity in ALS. Using RNA sequencing from a variety of sources (Supplementary Table S1), including enriched hiPSC-derived astrocytes harbouring mutations in *VCP, SOD1* and *C9orf72*, we found the loss of thousands of IR transcripts that correlates with increased abundance of their cognate spliced transcripts and are enriched for astrocyte reactivity processes. This decreased IR signature was recapitulated in both astrocytes stimulated with inflammatory cues to undergo reactive transformation as well as *in vivo* astrocytes following selective deletion of TDP-43, which itself triggered reactive transformation. We additionally found that a significant number of reactivity genes with reduced IR in ALS astrocytes also display increased translation. Using nucleocytoplasmic fractionation, we revealed that *VCP* mutant astrocytes have fewer IR transcripts in the nucleus, coupled with enhanced NMD of cytoplasmic IR transcripts. This was associated with increased cytoplasmic spliced mRNA and protein that are reactivity-related, suggesting nuclear-to-cytoplasmic translocation and translation of spliced reactivity transcripts. Cumulatively this work identifies a physiological and orchestrated IR programme in healthy astrocytes, which is lost in ALS and coincides with an increased abundance of reactivity regulators, thus potentially contributing to astrocyte reactive transformation.

## MATERIALS AND METHODS

### Human-induced pluripotent stem cell derived astrocytes

hiPSCs were maintained using standard protocols and were differentiated into astrocytes as described previously, generating highly enriched (>90%) populations of astrocytes (Supplementary Figure S1A) (8,11,48–51). hiPSCs were maintained on Geltrex (Life Technologies) with serum-free Essential 8 Medium media (Life Technologies), and passaged using EDTA. After neural conversion (7 days in a chemically defined medium containing 1 μM Dorsomorphin (Millipore), 2 μM SB431542 (Tocris Bioscience) and 3.3 μM CHIR99021 (Miltenyi Biotec), neural precursors were patterned for 7 days with 0.5 μM retinoic acid and 1 μM purmorphamine, followed by a 4-day treatment with 0.1 μM purmorphamine. After a propagation phase (60–120 days) with 10 ng/ml FGF-2 (Peprotech), astrocytes were terminally differentiated in presence of BMP4 (10 ng/ml, R&D) and LIF (10 ng/ml, Sigma-Aldrich) for 30 days. Informed consent was obtained from all patients and healthy controls in this study. Experimental protocols were all carried out according to approved regulations and guidelines by UCLH’s National Hospital for Neurology and Neurosurgery and UCL’s Institute of Neurology joint research ethics committee (09/0272).

### Nuclear and cytoplasmic RNA purification

Subcellular fractionation was achieved using the Ambion PARIS kit (ThermoFisher Scientific) following the manufacturer’s protocol. The cytosolic fraction was obtained by lysing cells in ice-cold cell fractionation buffer for 5 min, disrupting plasma membranes while leaving nuclear membranes intact. Lysates were centrifuged for 3 min at 500 × *g* at 4°C. Supernatant was further centrifuged at maximum speed at 4°C for 1 min, and the resulting supernatant was processed as the cytosolic fraction. Nuclear pellets from the first centrifugation step were gently washed with cell fractionation buffer and then lysed on ice for 30 min in 8 M Urea Nuclear Lysis Buffer. The resulting nuclear fraction was homogenized using a QIAshredder (Qiagen) to shred chromatin and reduce viscosity. Both lysis buffers were supplemented with 0.1 U/μl RiboLock RNase Inhibitor (ThermoFisher Scientific) and HALT Protease Inhibitor Complex (ThermoFisher Scientific).

### RNA isolation and RT-qPCR

The Promega Maxwell RSC simplyRNA cells kit including DNase treatment, alongside the Maxwell RSC instrument, was used for RNA extractions. The Nanodrop was used to assess RNA concentration and the 260/280 ratio, and the Agilent TapeStation was used to assess quality. All RNA samples have RIN value > 8.4. RT-qPCR was performed on cDNA generated from 50 ng DNaseI-treated total RNA using SuperScript® IV First-Strand Synthesis System (Invitrogen) and random hexamers, according to the manufacturers’ instructions. RT-qPCR reactions were performed in 10 µl volumes containing 1× SYBR® Green Mastermix (Bio-Rad) and 0.5 μM of the respective forward and reverse primers. Samples were amplified and analysed using the CFX96™ Real Time PCRMachine (Bio-Rad). Cycling conditions were: 50°C for 2 min, 95°C for 2 min, followed by 40 cycles at 95°C for 15 s, then 60 C for 60s. Samples were run in triplicate and all programs contained a melt curve and a no template control. The absence of contaminating gDNA was confirmed by PCR on negative RT samples. Fold change was calculated using the *C*_T_ method.

### RNA sequencing

Poly(A)+ selected reverse stranded RNA sequencing libraries were prepared from whole astrocyte and their nuclear and cytoplasmic fractions obtained from astrocyte differentiation from three control and two VCP mutant cell lines using the KAPA mRNA HyperPrep Library kit for Illumina®, with 50 ng of total RNA as input. Libraries were sequenced on a HiSeq 4000 platform at the Francis Crick Institute. One control sample failed quality control and was discarded. A total of 129 million 100 bp-long paired-end strand-specific reads were sequenced per sample split over five lanes. All libraries generated in this study had <1% rRNA, <1% mtDNA, >90% strandedness and >70% exonic reads (Supplementary Table S1).

### Proteomics

Protein samples from nuclear and cytoplasmic fractions were reduced, alkylated and acetone precipitated overnight. Each protein pellet was resuspended in 1 M guanidine hydrochloride and 100 mM HEPES. Proteins were tryptically digested overnight at 37°C with mixing. Digested samples were acidified then stored at −80°C. Each sample was split into triplicates and loaded onto prepared Evotips. Samples were analysed using Evosep 15 cm column and an orbitrap Fusion mass spectrometer operating in data-dependent acquisition mode. A 44-minute universal (OT/IT) method was used. Raw files were analysed with MaxQuant v1.6.12.0 using the LFQ algorithm against a 2020 SwissProt *Homo sapiens* protein database.

### Western blotting

Protein lysates were loaded onto NuPAGE, 4–12%, Bis-Tris protein gels (Invitrogen) and subjected to electrophoresis before being transferred onto nitrocellulose membrane (Biorad). Blocking was performed in PBS, 0.1% tween, 5% bovine serum albumin for 1 h at room temperature and incubated overnight with mouse anti α-Tubulin (1:1000, Sigma T5168) and rabbit anti-histone 3 (1:1000, Abcam ab179). Following washes, membranes were incubated for 1 h at room temperature with fluorescent secondary anti-rabbit and anti-mouse antibody (1:10000, LI-COR IRDye). Membranes were imaged using Odyssey Fc Imaging System (LI-COR).

### Statistical analyses

Raw mRNA sequencing reads were aligned to hg38 human reference genome using splice-aware aligner, STAR v2.6.1 (52). Aligned reads were quantified with HTSeq v0.12.4 (53) using intersection-strict mode to enable counting of spliced transcripts at gene-level based on Ensembl GRCh 38.99 annotation. Detailed quality control of the raw RNAseq was assessed utilizing the nf-core/rna-seq pipeline (54) (Supplementary Tables S1, S3). Differential gene expression was measured using DESeq2 (55) in R v4.0.3. Results were generated by comparing the mutant (or treated, or cytoplasmic fraction) versus control (or untreated, or nuclear fraction) and a gene was considered significantly differentially expressed if false discovery rate (FDR) < 0.05.

**Figure 1.**
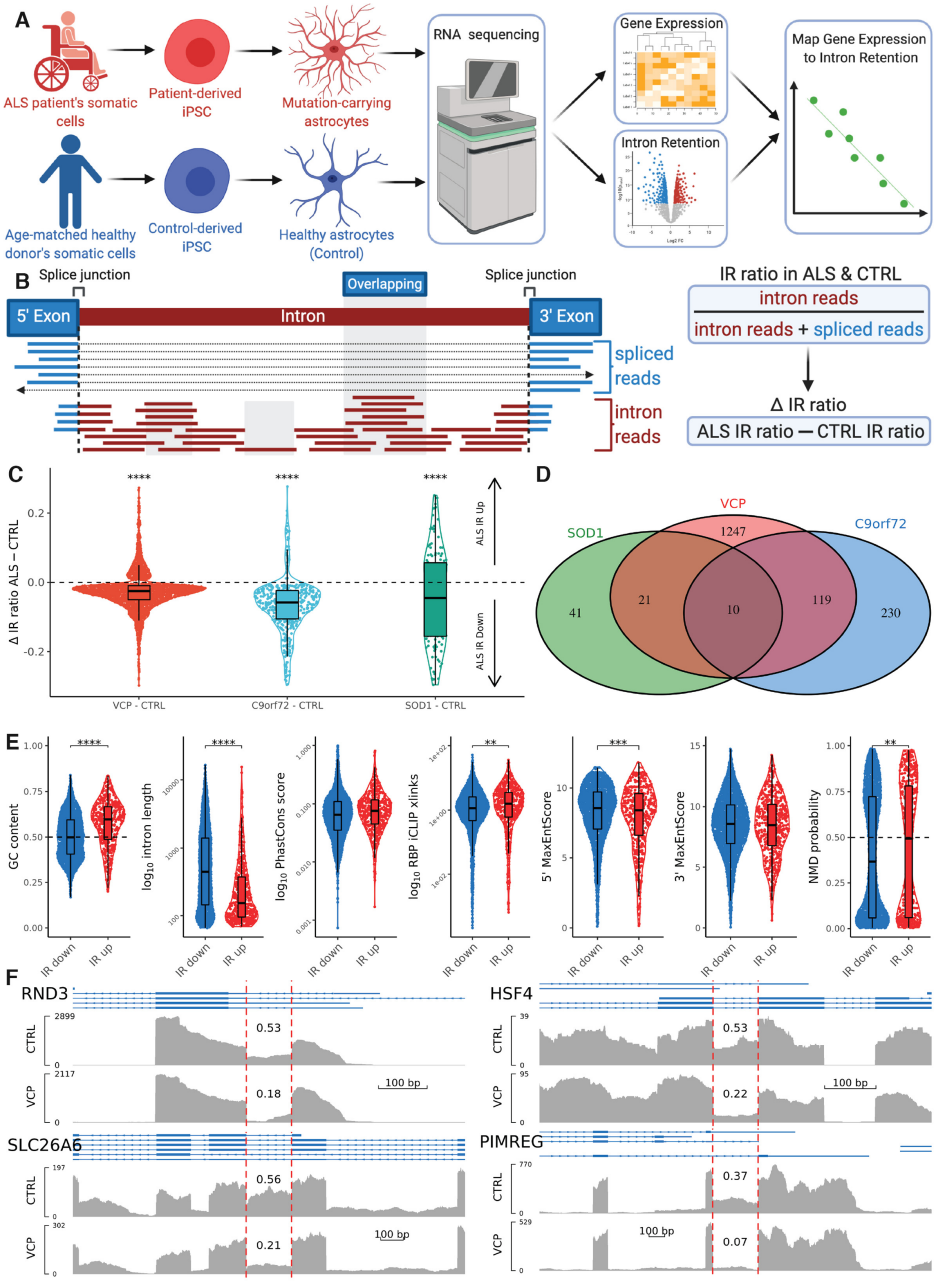
RNA-sequencing reveals loss of intron retention in ALS astrocytes. (**A**) Schematic depicting human-induced pluripotent stem cells (hiPSC) astrocyte differentiation and fractionation in ALS (red) and control (blue). Astrocytes were subjected to RNA-sequencing and were analysed for differential gene expression and alternative splicing, with a focus on intron retention. We then mapped the spliced-transcript expression and intron retention values of each gene. See Supplementary Figure S1A for stepwise differentiation strategy of iPSC-derived astrocytes. Two hiPSC lines were obtained from two ALS patients with VCP mutations (R155C and R191Q) and two healthy controls. (**B**) Schematic of IR quantification and differential IR calculation between mutant and control. Median intron coverage is calculated after excluding non-unique multi-mapping reads, overlapping exons (blue) and outliers (highest and lowest 30%) depicted by the shaded regions. Exon and intron read abundance is normalized for feature length. IR ratio is calculated as intron abundance divided by the sum of intron and spliced abundance. Reliable IR event expression is defined as (1) spliced reads mapping across the 3′ and 5′ flanking exons + intron reads > 10 and (2) > 4 reads from across the 3′ and 5′ flanking exons. Delta IR ratio is calculated as mutant minus control IR ratio. Adapted from Wong *et al.* (36) and Middleton *et al.* (58). (**C**) Violin plot showing delta IR ratio in ALS mutants minus control across differential intron retention events. Intron retention was significantly downregulated in VCP (red, *n* = 2309), C9orf72 (light blue, *n* = 448) and SOD1 (green, *n* = 139) astrocytes versus CTRL (one-sample *t*-test, **** represents *P* < 0.0001). (**D**) Venn diagram showing the number of overlapping genes with decreased intron retention events in VCP (red), C9orf72 (blue) and SOD1 (green). Gene overlap exhibiting decreased IR between SOD1 and VCP datasets was 31/76 (40.1%, *P* = 1.8 × 10^−18^, hypergeometric test). C9orf72 and VCP overlap was 129 / 347 (37.2%, *P* = 1.8 × 10^−67^. Overlapping all three mutants chi-squared *P* = 0.005. (**E**) Violin plots showing intron characteristics (GC content; intron length; phastCons conservation score; iCLIP crosslinking with RBPs; 5′ and 3′ Maximum Entropy Scores); and nonsense mediated decay (NMD) probability of upregulated versus downregulated retained introns in ALS mutants (VCP, C9orf72 and SOD1) versus control astrocytes (IR down, *n* = 2339; IR up, *n* = 490; **** represents adjusted *P* values from Wilcoxon test < 0.0001, *** < 0.001, ** *P* < 0.01). For intron characteristics of individual ALS mutants see Supplementary Figure S4B. (**F**) Coverage plots of reads per million from VCP mRNA sequencing reads for RND3, SLC26A6, HSF4 and PIMREG genes. Within each are alignment tracks for CTRL (top) and VCP (bottom) astrocytes. Ensembl transcript annotation tracks are shown above the alignment tracks with exons (thick blue boxes) and introns (thin blue arrowed lines). Vertical red dashed lines show the retained intron of interest and the IR ratio is labelled above the retained intron. *Y*-axis limits for each track are auto-adjusted to the peak exon coverage so that the target feature fills the view and enables easier visual comparison of IR ratios between conditions.

Alternative splicing was quantified using both a coverage based tool, *VAST-TOOLS* (56) and a splice-junction tool, *SGSeq* (57). IR focused analysis was quantified using *IRFinder*, (58) which measures IR levels using the IR ratio metric (intron read depth divided by the sum of the intron and flanking exon read depth, Figure 1B). *IRFinder* addresses specific peculiarities in IR quantification including: low complexity regions, non-poly(A)+ RNA and DNA contamination, and overlapping exons. To improve consistency in the quantification of IR only ‘clean’ events are included, filtering out spurious IR events with reliability warnings LowCover (spliced reads mapping across the 3′ and 5′ flanking exons + intron reads > 10) and LowSplicing (> 4 reads from across the 3′ and 5′ flanking exons) in the IRFinder-IR-dir.txt output. Differential IR was quantified using *analysisWithLowReplicates.pl* from *IRFinder* between mutant and control, using the Audic and Claverie test and threshold of *P* < 0.05 (uncorrected for multiple testing). To assess the relationship between differential gene expression and IR, we assigned the log_2_foldchange in its gene expression (mutant versus control) to the ΔIR ratio for each reliable differential IR event. To identify enriched functional pathways, Gene Ontology (GO) enrichment was performed using *g:Profiler2* (59). Significantly over-represented (FDR < 0.05) up and downregulated IR genes were grouped according to their differential gene expression directional change and these subsets were used as input, with all measured genes used as background. In the barcharts the top significant GO terms were manually curated by removing redundant terms. Gene set enrichment analysis (GSEA) was performed using the fgsea R package (60) on the transcriptomic signature gene sets for NMD components (61) and Liddelow *et al.* reactivity genes (24). Overlapping gene lists between datasets were tested using the Fisher’s exact test. Similarly, Liddelow *et al.* reactivity genes with reduced IR were tested for overlap with genes increased in expression using the Fisher’s exact test. For Liddelow *et al.* genes with multiple reliable ΔIR ratios, we calculated the genes mean ΔIR ratio, normalizing for each retained introns length.

Features of retained and spliced introns (length, GC content, PhastCons conservation score) were analysed as reported previously (36,42). To identify RBPs that bind to aberrantly retained introns, we examined iCLIP data for 21 RBPs (62), and eCLIP data from HepG2 cells for 112 RBPs available from ENCODE (63). Relative enrichment for each of the RBPs was obtained by calculating the proportion of crosslink events mapping to retained introns compared with non-retained introns of the same genes. Maximum Entropy splice site scoring software (MaxEntScan) was used to predict the splice site strength for the 5′ splice sites (5′ ss) and 3′ splice sites (3′ ss) (64). The 5′ss score uses 9-bases (last 3 bases of the upstream exon and first 6 bases of the intron), whereas the 3′ss score uses 23-bases (last 20 bases of intron and first 3 bases of the downstream exon). We determined the MaxEntScan score for retained introns, using all 5′ ss (exon–intron junctions) and 3′ ss (intron–exon junctions) of annotated introns, using the *GencoDymo* R package. NMD probability was predicted by searching for premature termination codons in the open reading frame of transcripts containing retained introns, using *GeneStructureTools* and *notNMD* bioconductor packages. Differential transcript-level isoform expression analysis was performed with Kallisto (65) and transcript biotypes were annotated using the Ensembl biotype classification to enable comparison between NMD and protein coding annotated transcripts.

For comparison with *SOD1* and *C9orf72* mutant hiPSC astrocytes, we downloaded RNAseq data from Tyzack *et al.* (8) (GSE102902 and GSE99843) and Birger *et al.* (66) (GSE142730). SOD1^G37R^ mouse spinal cord astrocyte TRAP-Seq was downloaded from Sun *et al.* (67) (GSE74724). For comparison with hiPSC stimulated reactive astrocyte dataset, we downloaded bulk RNAseq from Barbar *et al.* (27) (synapse ID: 21861229). For TDP-43 deleted mouse astrocytes, we accessed bulk RNAseq from Peng *et al.* (68) (GSE156542). RNAseq from these studies were processed using the same pipeline as described above and quality control metrics are reported in Supplementary Table S1. Sequencing depth was on average ∼50 million reads per sample, which ranged from 10 million in SOD1 to 129 million in VCP mutant astrocytes. Lower sequencing depth limits the ability to detect IR; however, using the stable IRFinder algorithm, we were able to identify reliable IR events even at 10 million albeit at lower absolute numbers (Supplementary Figure S3) (8,69). Mass spectrometry proteomics data were analysed using DEP package (v1.11.0) (70) on MaxQuant results. Data were filtered, normalized and imputed using default parameters. Differential enrichment analysis was performed using protein-wise linear models combined with Bayes statistics that utilizes *limma*. A protein was considered significantly differentially expressed when *P* < 0.05 (uncorrected for multiple comparisons). Schematics were created with *BioRender.com*. All error bars shown represent either the standard error of the mean (SEM) or 1.5 times the interquartile range, from independent experiments.

## RESULTS

### Intron retention is decreased in ALS astrocytes across *VCP*, *SOD1* and *C9orf72* mutants

To identify transcriptome-wide changes in ALS astrocytes, we performed RNA sequencing on poly(A)+ selected mRNA libraries isolated from highly enriched and functional hiPSC-derived astrocytes using our previously established platform (Supplementary Figure S1A) (8,11,49–50,71). Specifically, we first examined RNA sequencing from two lines derived from two patients with ALS-causing *VCP* gene mutations (p.R155C and p.R191Q) and two healthy control lines (Figure 1A) (8,11,49). To improve confidence in detecting differential splicing, libraries were deeply sequenced to ∼130 million reads per sample (Supplementary Table S2). Principal component analysis and unsupervised hierarchical clustering demonstrated that samples segregated based on genotype (Supplementary Figure S1B–D). We first defined differences between *VCP* mutant and control astrocyte gene expression patterns and found 170 differentially expressed genes (FDR < 0.05), which is 11-fold more than what we previously established in terminally differentiated *VCP* mutant motor neurons (41). Differentially expressed genes were enriched for developmental, structural and nervous system processes (Supplementary Figure S1E and Supplementary Table S3).

We sought to examine alternative splicing in hiPSC-derived astrocytes, comparing those carrying *VCP* mutations to control counterparts. After examining all modes of alternative splicing and finding that only IR was significantly different between *VCP* mutant and control astrocytes (Supplementary Figure S2A and B), we focused further on IR quantification (Figure 1B) (58). Across both conditions, we found 16,317 reliable unique retained introns (IR ratio > 0.1 i.e. intron abundance is ≥ 10% of the sum of intron + spliced abundance); however, there were 14.1% fewer retained introns in *VCP* mutant compared with control (12,169 versus 14,162, respectively, Supplementary Figure S3A). Differential IR comparison revealed 2,309 introns with reliable coverage from 1,699 genes that were retained at significantly different levels (*P* < 0.05, Audic and Claverie test, Supplementary Figure S3B), with 1,885 (81.6%) displaying decreased IR in *VCP* (Figure 1C, 1F; *P* = 2.39 × 10^−68^), confirming reduced IR in *VCP* mutant astrocytes.

To assess whether IR was also reduced in other ALS mutant astrocytes, we analysed published *C9orf72* (66) and *SOD1* (8) mutant astrocyte RNA sequencing datasets (*C9orf72* dataset: two mutant and two control lines; *SOD1* dataset: one mutant and two control lines) (Supplementary Table S1). In both datasets we identified dramatically less retained introns in ALS mutants compared with control (*C9orf72*: 7,925 versus 14,937 [47% less, Supplementary Figure S3C]; *SOD1*: 3,602 versus 7,842 [54% less, Supplementary Figure S3E] respectively, Figure 1C). In *C9orf72* astrocytes, 448 introns in 376 genes were significantly differentially retained, of which 93% (416/448) were decreased in *C9orf72* relative to control (Supplementary Figure S3D). In *SOD1* mutant astrocytes, 139 introns in 131 genes showed significant differential retention of which 59% (82/139) were reduced in SOD1 mutants compared with control (Supplementary Figure S3F). These findings from across five ALS mutant and six control lines, when taken together, strongly indicate that decreased IR in astrocytes is common across diverse genetic forms of ALS.

To investigate whether ALS mutant astrocytes exhibit decreased IR within the same genes, we overlapped genes with IR events between *VCP, C9orf72* and *SOD1* mutants. Comparing genes exhibiting decreased IR between *VCP* and *C9orf72* datasets, revealed a significant overlap of 129 / 347 (37.2%, *P* = 2.0 × 10^−118^, Fisher’s exact test; Figure 1D). Likewise, *VCP* and *SOD1* mutants, shared 31 / 76 (40.1%, *P* = 9.0 × 10^−31^) genes with decreased IR (Supplementary Table S4). Ten genes with decreased IR were common to all three mutations (chi-squared *P* = 0.005), of which six are relevant to reactivity: *FLNA, PLOD3, MRC2*, *ACTN4, GPS2* and *MCAM* (28,72). This significant overlap between independent ALS datasets recapitulates our observation in *VCP* mutant astrocytes, suggesting that diminished IR of astrocyte reactivity genes is generalizable across ALS mutations.

Consistent with prior studies, retained introns in all three datasets (i.e. *VCP, C9orf72* and *SOD1* mutant ALS astrocytes) exhibited significantly shorter lengths, higher GC content, higher conservation score, higher predicted binding affinities to RNA-binding proteins (RBPs) and lower splice site strength compared with spliced introns (Supplementary Figure S4A) (36,73–74). Introns with significantly decreased retention in any of the three ALS mutants exhibited longer lengths, lower GC content, and lower RBP-binding affinities but similar conservation scores compared with gained introns (Figure 1E) (63,75). Although 3′ splice site strengths were similar between increased and decreased IR events, the 5′ splice site strengths were higher in decreased IR events, consistent with increased activity of the spliceosome machinery in ALS astrocytes (64). Scanning for premature termination codons (PTC) within the open reading frame of intronic sequences revealed that the probability of NMD was significantly lower in IR transcripts decreased in ALS compared to IR transcripts increased in ALS. These observations were generally consistent between VCP, C9orf72 and SOD1 mutants (Supplementary Figure S4B) and confirm previous studies demonstrating that *cis* features define an ‘IR code’ (36,42,45).

### Decreased intron retention and increased expression of astrocyte reactivity regulators is generalizable across ALS astrocytes

**Figure 2.**
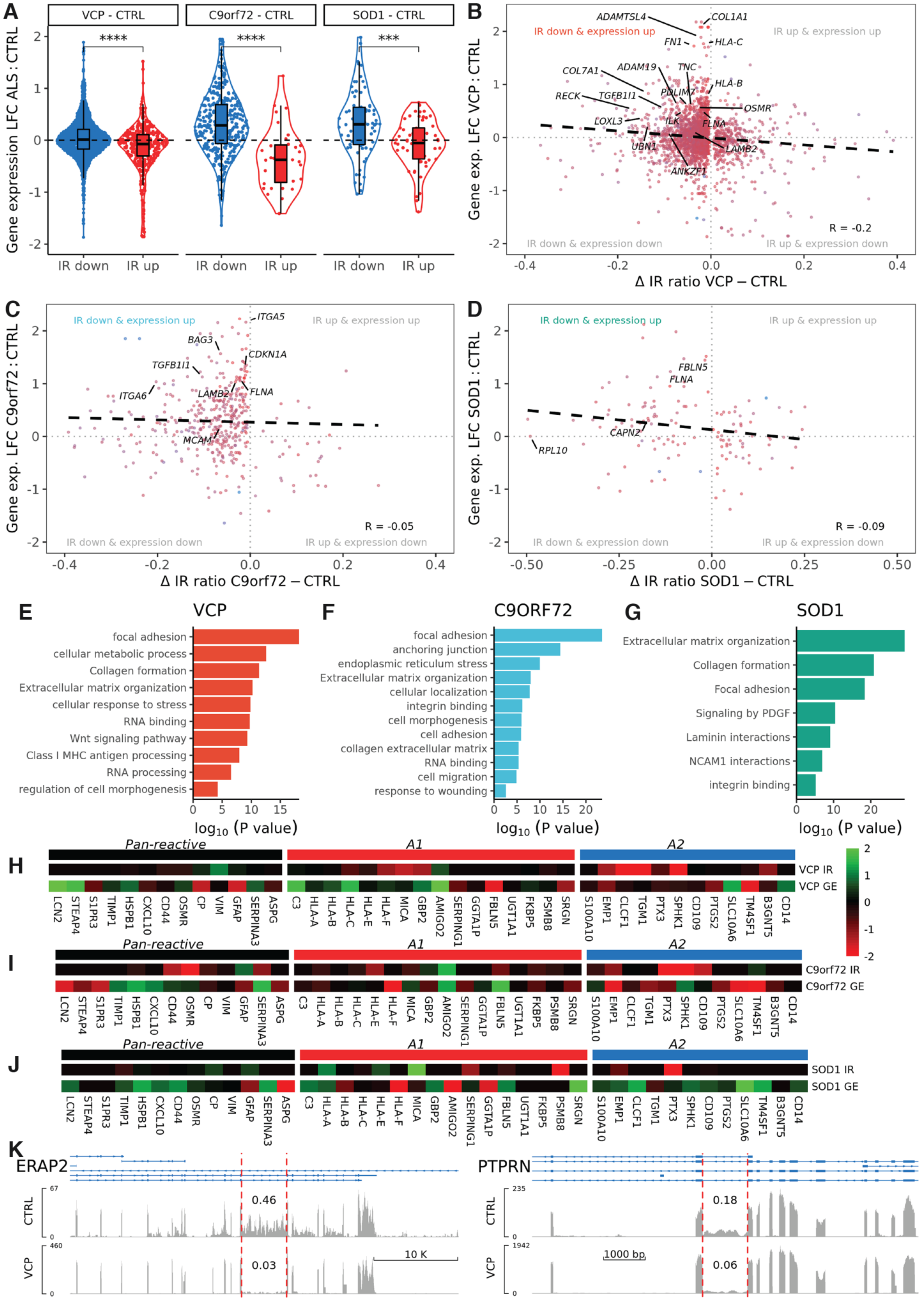
Intron retention is associated with increased expression of genes involved with astrocyte reactivity. (**A**) Violin plots showing log2 fold change in gene expression (*Y*-axis) in downregulated IR events (blue) and upregulated IR events (red) for VCP (IR down, *n* = 1885; IR up, *n* = 424), C9orf72 (IR down, *n* = 416; IR up, *n* = 32), SOD1 (IR down, *n* = 82; IR up, *n* = 57). Student’s *t*-test was used to determine significance (**** indicates *P* < 0.0001, *** *P* < 0.001). (**B-D**) Scatterplots of the Δ IR ratio (ALS minus control, *X*-axis) against log2 fold change in gene expression (*y*-axis) in VCP (B), C9ORF72 (C) and SOD1 (D) versus control astrocytes. Points are coloured by mean gene expression across mutant and control (red = high, blue = low expression). Black dashed line indicates linear regression correlation (VCP pearson correlation, *R* = −0.20; C9orf72 *R* = −0.05, SOD1 *R* = −0.09). Grey dotted lines indicate the points of no difference between ALS versus control astrocytes for IR ratio (*X*-axis) and gene expression (*Y*-axis). Reactivity related transcripts with IR down and expression up are labelled. See Figure 2E–G for processes enriched amongst genes in the IR down and expression up quadrant. For the three remaining quadrants see Supplementary Figure S5A–C. (**E–G**) Bar graphs showing significantly over-represented functional categories (FDR < 0.05) determined by gene ontology analysis of transcripts with reduced IR and increased expression in VCP (E), C9orf72 (F), SOD1 (G) versus control astrocytes. (**H–J**) Heatmaps of pan-reactive (black), A1 specific (red), and A2 specific (blue) markers from Liddelow *et al.*, 2017 (24). IR and gene expression (GE) data represent the log2 fold change in VCP (J), C9orf72 (K), SOD1 (L) versus control astrocytes. (**K**) Coverage plots of reads per million from VCP mRNA sequencing reads for differentially expressed genes ERAP2 and PTPRN genes. Within each are alignment tracks for CTRL (top) and VCP (bottom) astrocytes. Ensembl transcript annotation tracks are shown above the alignment tracks with exons (thick blue boxes) and introns (thin blue arrowed lines). Vertical red dashed lines show the retained intron of interest and the IR ratio is labelled above the retained intron. *Y*-axis limits for each track are auto-adjusted to the peak exon coverage so that the target feature fills the view and enables easier visual comparison of IR ratios between conditions.

To understand the consequences of decreased IR in ALS astrocytes on gene expression, for each gene we mapped levels of IR to expression (Figure 1A). Previous studies indicate that decreased IR can lead to increased gene expression through avoiding NMD (30,76–77). Comparing changes in IR with changes in gene expression, we found that genes with decreased IR had higher expression compared with genes with increased IR in *VCP* mutant versus control astrocytes (*t*-test *P* = 2.6 × 10^−11^; Figure 2A). Correlating changes in IR with changes in gene expression between *VCP* mutant and control revealed a negative association (Pearson *R* = −0.20, Figure 2B), consistent with a relationship between transcripts with decreased IR and increased expression of their spliced isoforms. Of 817 genes with increased expression and exhibiting a reliable IR event, 702 (85.9%) exhibited decreased IR in *VCP* mutant astrocytes. Restricting to genes with significantly increased expression in *VCP* mutant astrocytes (FDR < 0.05) and a reliable IR event, revealed that all four were also significantly decreased in IR (*ERAP2, PTPRN, IL20RB*, and *KCNE4;* Figure 2K). Using gene ontology analysis, we found genes with reduced IR and increased expression in *VCP* mutant astrocytes were over-represented in pathways associated with cell adhesion (e.g. *TGFB1I1, COL7A1, TNC, RECK, ERAP2*), stress response (e.g. *STX4, PTPRN, SLC26A6, NUP199, HSF4*) and immune activation (e.g. *LOXL3, NFKB2, HLA-B* and *HLA-C*) - processes involved in astrocyte reactivity (Figure 2E) (78–80). Conversely, genes with increased IR and increased expression in *VCP* mutant astrocytes were enriched for enzyme activity rather than reactivity related terms (Supplementary Figure S5A). Of the Liddelow *et al.* (24) astrocyte reactivity genes with a reliable IR event, 21/31 were decreased in IR and 15/31 were increased in expression, while 10/31 exhibited both (32%; overlap *P* = 0.056; Figure 2H). Taken together, these data suggest an inverse relationship between IR and expression, with *VCP* mutant astrocytes exhibiting decreased IR and increased expression of genes regulating astrocyte reactivity.

Similar to *VCP* mutant astrocytes, for both *C9orf72* and *SOD1* mutants, genes with decreased IR exhibited significantly increased gene expression, whereas genes with increased IR correlated with decreased expression (C9orf72: *P* = 4.5 × 10^−8^; SOD1: *P* = 1.5 × 10^−4^; Figure 2A). Additionally, both *C9orf72* and *SOD1* mutants exhibited a negative correlation between changes in IR and changes in gene expression (*C9orf72R* = -0.05; *SOD1R* = -0.09; Figure 2C and D). Gene ontology analysis revealed transcripts with decreased IR and increased expression were also over-represented in astrocyte reactivity pathways (Figure 2F and G; Supplementary Figure S5B and C). Of the Liddelow *et al.* (24) astrocyte reactivity genes exhibiting a reliable IR event, we observed decreased IR and increased expression in 44% (11/25, overlap *P* = 0.0002) for *C9orf72* mutants and 42% (8/19, overlap *P* = 0.0002) for *SOD1* mutants (Figure 2I and J). Comparing genes exhibiting decreased IR and increased expression in *SOD1* mutants with *VCP* mutants revealed a significant overlap of 18/55 (33%, *P* = 1.1 × 10^−21^, hypergeometric test; Supplementary Figure S5D). Likewise, of the 251 genes exhibiting decreased IR and increased expression in *C9orf72*, 70 (28%) were also found in *VCP* mutants (*P* = 2.7 × 10^−75^; Supplementary Table S5). Six genes with decreased IR and increased expression were common to all three mutations (chi-squared *P* = 0.008), of which four are directly relevant to reactivity: *FLNA, PLOD3, MRC2* and *ACTN4* (28,72). This significant overlap between independent ALS datasets recapitulates our observation in *VCP* mutant astrocytes, demonstrating that diminished IR and enhanced expression of astrocyte reactivity genes are generalizable across ALS mutations.

### Stimulating reactive transformation using inflammatory cues recapitulates decreased intron retention and increased expression of reactivity genes

**Figure 3.**
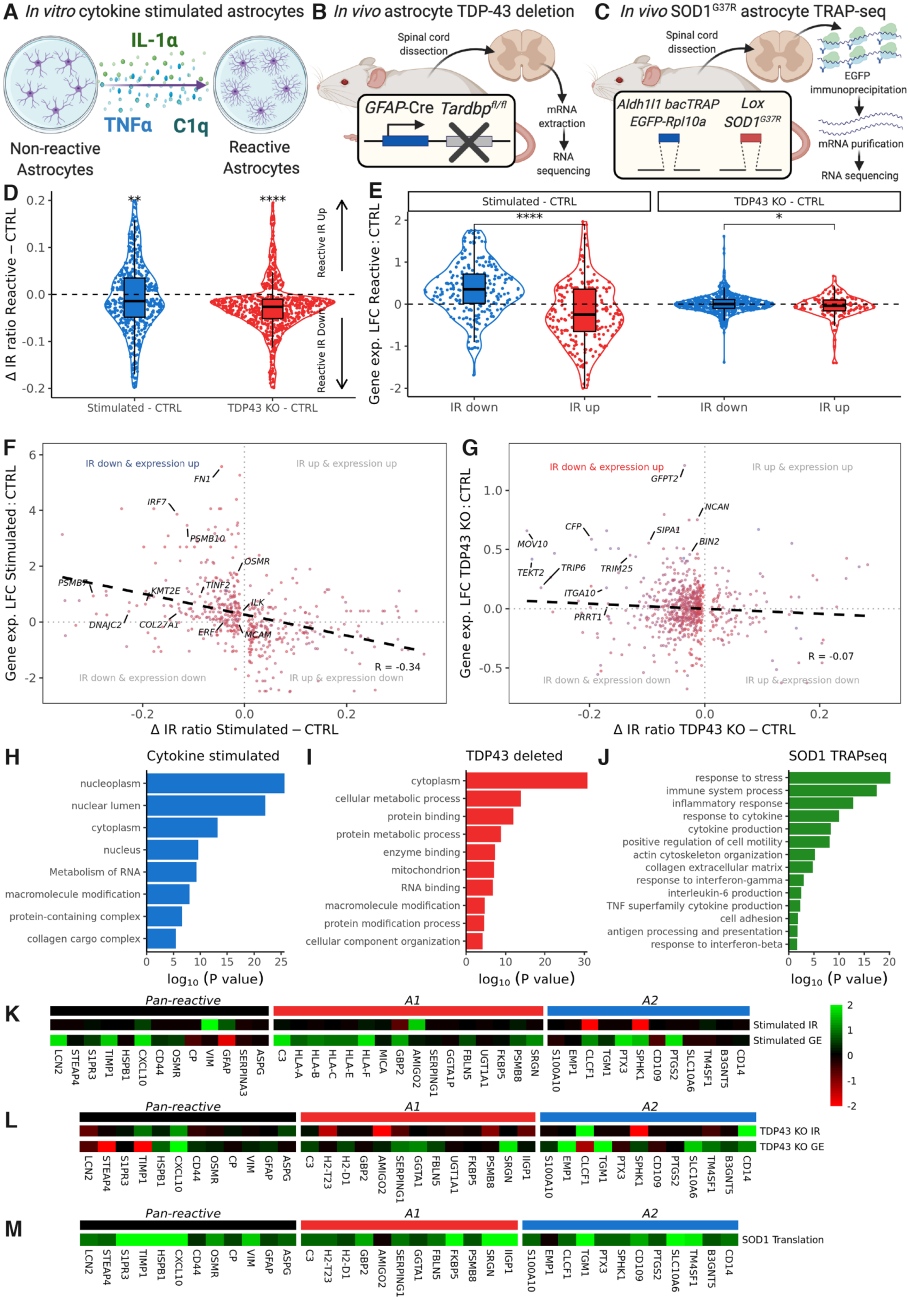
Loss of intron retention and increased expression of astrocyte reactivity genes is in reproduced cytokine stimulation of astrocytes *in vitro* and astrocyte TDP-43 deletion *in vivo*, which coincides with increased translation in *SOD1^G37R^* astrocytes. (**A–C**) Schematics depicting (A) *in vitro* inducing astrocyte reactivity with inflammatory cues (TNFα, IL-1α, C1q) in hiPSC-derived control astrocytes, (B) *in vivo* astrocyte-specific TDP-43 deletion using conditional GFAP-Cre recombinase promoter. Spinal cord was dissected and mRNA extracted before RNA sequencing, and (C) ALS SOD1^G37R^ mutant mouse with astrocyte-specific (Aldh1l1) bacTRAP reporter, which expresses the EGFP-tagged ribosome protein (Rpl10a) within astrocytes. Polyribosome-associated mRNAs undergoing translation are then isolated by EGFP immunoprecipitation before mRNA purification and RNA translatome sequencing. (**D**) Violin plot showing delta IR ratio in reactive astrocytes (blue, left, *n* = 578) and TDP43 deletion (red, right, *n* = 700) minus control across differential intron retention events. Intron retention was significantly downregulated in both cytokine stimulated astrocytes and TDP43 deleted astrocytes (one-sample *t*-test, **** represents *P* < 0.0001 and ** *P* < 0.01). (**E**) Violin plots showing log2 fold change in gene expression (reactive state versus control, *y*-axis) in downregulated IR events (blue) and upregulated IR events (red) for cytokine stimulated (IR down, *n* = 304; IR up, *n* = 274) and TDP-43 deleted (ko, knockdown) (IR down, *n* = 581; IR up, *n* = 119). Student’s *t*-test was used to determine significance (**** indicates *P* < 0.0001, * *P* < 0.05). (**F** and**G**) Scatterplots of the Δ IR ratio (reactive state minus control, *x*-axis) against log2 fold change in gene expression (*y*-axis) in (F) cytokine stimulated and (G) TDP43 deleted versus control astrocytes. Points are coloured by mean gene expression across mutant and control (red = high, blue = low expression). Black dashed line indicates linear regression correlation (cytokine stimulated Pearson correlation *R* = −0.34; TDP43 deleted *R* = −0.07). Reactivity related genes with IR down and expression up are labelled. See Figure 3H–I for processes enriched amongst genes in the IR down and expression up quadrant. (**H** and**I**) Bar graphs showing significantly over-represented functional categories (FDR < 0.05) determined by gene ontology analysis of genes with reduced IR and increased gene expression in cytokine stimulated (H) and TDP43 deleted (I). See Supplementary Figure S5E and F for the other IR and expression categories. (**J**) Bar graph showing curated significantly over-represented functional categories (FDR < 0.05) determined by gene ontology analysis of genes with increased translation in SOD1^G37R^ versus control mouse spinal cord astrocytes. See Supplementary Figure S5G for genes decreased in translation. (**K** and**L**) Heatmaps of pan-reactive (black), A1 specific (red) and A2 specific (blue) markers from Liddelow *et al.*, 2017 (24). IR and gene expression (GE) data represent the log2 fold change in cytokine stimulated (K) and TDP43 deleted (L) versus control astrocytes. (**M**) Heatmap of pan-reactive, A1 specific and A2 specific markers in astrocyte translatome data log2 fold change in SOD1^G37R^ versus control samples.

Having established that astrocytes with a range of familial ALS mutations exhibit reduced IR, we sought to determine if artificially stimulating astrocyte reactivity with inflammatory cytokines (TNFα, IL-1α and C1q) was sufficient to induce the same effect (Figure 3A) (27). By analysing RNA sequencing from Barbar *et al.* hiPSC-derived astrocytes stimulated to undergo reactive transformation (three reactive and three untreated lines) (27), we identified 11% less retained introns in astrocytes stimulated with TNFα, IL-1α and C1q compared with untreated (basal) astrocytes (2,143 versus 2,402, respectively; Supplementary Figure S3G). Differential IR analysis revealed 578 introns from 430 genes that were retained at significantly different levels, with 304 (53%) being reduced in cytokine-stimulated astrocytes (Figure 3D, S3H; one-sample *t*-test *P* = 0.001), indicating reduced IR in hiPSC-derived reactive astrocytes. Of the 240 genes with decreased IR in cytokine-stimulated astrocytes, 86 (35.8%) also exhibited decreased IR in the ALS astrocytes (overlap *P* = 3.8 × 10^−71^; Supplementary Table S4). Taken together, we find that IR is prevalent in healthy quiescent hiPSC-derived astrocytes but that ALS astrocytes (with *VCP, SOD1* and *C9orf72* mutations) as well as astrocytes stimulated using TNFα, IL-1α, and C1q, share a common decreased IR signature.

By comparing gene expression with IR after cytokine-stimulation in control astrocytes, we observed that genes with decreased IR in stimulated astrocytes had significantly higher gene expression (*P* < 2.22 × 10^−16^; Figure 3E). Furthermore, IR events with greater decreases in the IR ratio exhibited larger increases in gene expression (*R* = −0.34, Figure 3F). Enrichment analysis of genes with decreased IR and increased expression reveals that they are over-represented in pathways associated with cellular compartments, RNA metabolism and collagen processing, analogous to processes implicated in ALS astrocytes (Figure 3H). Of the Liddelow *et al.* (24) reactivity genes with a reliable IR event, 11/32 (34%) exhibited both decreased IR and increased expression (overlap *P* = 0.005, Figure 3K). Comparing genes with decreased IR and increased expression between stimulated astrocytes and ALS astrocytes revealed an overlap of 45/189 (23.8%, overlap *P* = 4.2 × 10^−40^), of which 17 are directly relevant to astrocyte reactivity (Supplementary Table S5) (8,26,81). This indicates that stimulating astrocyte reactivity via established pro-inflammatory cues reproduces reduced IR and increased expression in similar reactivity related genes as ALS astrocytes.

### *In vivo* astrocytes with selective deletion of TDP-43 exhibit decreased intron retention

To further explore the molecular causes associated with decreased IR in ALS astrocytes, we next investigated the role of TDP-43 in this context given its salience as a pathological hallmark in motor neurons. To address this, we leveraged RNA sequencing data from a recent *in vivo* study that demonstrated A1-like reactive transformation in astrocytes upon TDP-43 depletion (68). We sought to determine if this mouse spinal cord astrocyte-specific *TARDBP* knockout (TDP-43 deletion) influenced IR (four knockout and four control mice; Figure 3B). This revealed 11% less retained introns in TDP-43 deleted astrocytes compared with control (12,716 versus 14,338 respectively; Supplementary Figure S3I). Differential IR analysis revealed 700 introns from 643 genes that were retained at significantly different levels (*P* < 0.05, Supplementary Figure S3J), with 581 (83%) being decreased (Figure 3D; one-sample *t*-test *P* = 1.10 × 10^−20^), indicating that astrocyte TDP-43 deletion induces a global reduction in IR. Comparing genes with decreased IR between TDP-43 deleted astrocytes and ALS astrocytes revealed an overlap of 139/525 (26.5%, *P* = 1.2 × 10^−92^; Supplementary Table S4). These results indicate that astrocyte TDP-43 is required to maintain the abundant physiological IR levels and the loss of TDP-43 may trigger the decrease in IR we have revealed in ALS astrocytes.

By comparing gene expression with IR in TDP-43 deleted astrocytes, we observed that genes with decreased IR had significantly higher gene expression (*P* = 0.01, Figure 3E). Consistent with the other datasets, we found a negative correlation between changes in IR and changes in gene expression (*R* = −0.07; Figure 3G). Also similar to both ALS and cytokine-stimulated astrocytes, gene ontology analysis showed that they are enriched in metabolic processes, cellular compartments and RNA binding (Figure 3I). Of the Liddelow *et al.* (24) astrocyte reactivity genes with a reliable IR event, 12/31 (39%) were decreased in IR and increased in expression (overlap *P* = 0.01, Figure 3L). Comparing genes with decreased IR and increased expression between the TDP-43 deleted astrocytes and the ALS astrocytes revealed an overlap of 46/269 (17.1%, *P* = 4.6 × 10^−34^), of which 19 are directly relevant to astrocyte reactivity (Supplementary Table S5) (26). This suggests that astrocyte TDP-43 depletion recapitulates reduced IR and increased expression in similar reactivity related genes as ALS astrocytes.

### Reactivity genes with decreased IR in ALS astrocytes are translated in a *SOD1* mouse model of ALS

To determine whether decreased IR in ALS astrocytes influences translation of astrocyte reactivity genes, we examined a translating ribosome affinity purified RNA sequencing (TRAP-Seq) dataset from Sun *et al.* (Figure 3C) (67). *SOD1^G37R^* mutant mouse spinal cord astrocytes were isolated and mRNAs bound to ribosome subunits were sequenced enabling interrogation of these translating mRNAs (four *SOD1^G37R^* and six control samples). In concordance with Sun *et al.*, we found that *SOD1^G37R^* astrocytes exhibit upregulation of inflammatory pathways (Figure 3J and Supplementary Figure S5G) as well as established astrocyte reactivity genes (24) (enrichment *P* = 7.3 × 10^−10^; Figure 3M). Comparing genes with significantly increased translation in Sun *et al.* (FDR < 0.05) with those with decreased IR in ALS astrocytes, revealed 44/429 (9%, *P* = 9.2 × 10^−^^14^) overlapping genes, of which 32 are directly relevant to astrocyte reactivity, including *OSMR*, *FBLN5, VIM* and *PTX3* (Supplementary Table S4) (26). Additionally, of the genes exhibiting decreased IR in stimulated reactive astrocytes, 6/223 (*P* = 0.01) displayed increased translation in *SOD1^G37R^* mouse astrocytes, of which all eight were decreased in IR in ALS astrocytes. Similarly, in the astrocyte TDP-43 deleted mouse, 17 genes with decreased IR were increased in translation in *SOD1^G37R^* astrocytes (*P* = 4.4 × 10^−^^7^), of which 7 were also decreased in IR in ALS mutants. Collectively, these data suggest that decreased IR in ALS astrocytes is correlated with translation of reactivity genes.

### Decreased nuclear intron retention is associated with increased cytoplasmic spliced reactivity transcripts and protein in *VCP* mutant astrocytes

**Figure 4.**
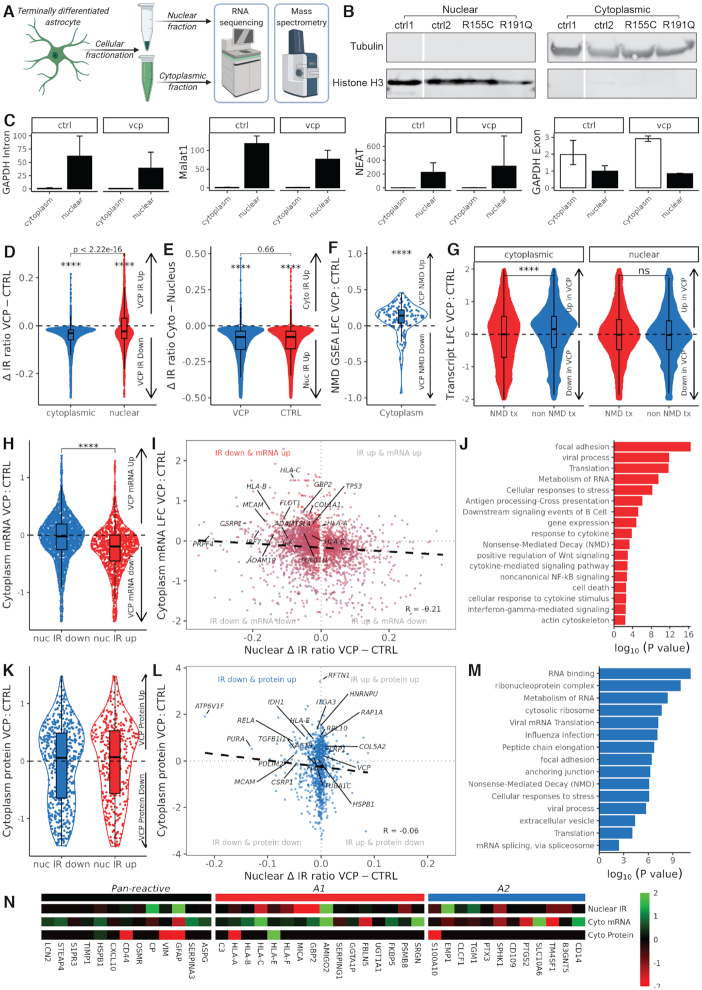
Decreased nuclear intron retention coincides with increased cytoplasmic spliced reactivity transcripts and protein in *VCP* mutant astrocytes. (**A**) Schematic of astrocyte fractionation into nuclear and cytoplasmic compartments followed by RNA sequencing and mass spectrometry. (**B**: western blot of histone 3 (bottom, nuclear marker) and Tubulin (top, cytoplasmic marker) in nuclear (left) and cytoplasmic (right) protein from control (ctrl1, ctrl2) and VCP (R155C, R191Q) astrocytes. Gaps indicate non-adjacent samples run on the same blot. (**C**) RT-qPCR of nuclear markers intronic GAPDH, Malat1, NEAT and cytoplasmic marker exonic GAPDH transcript fold enrichment in nuclear and cytoplasmic fractions from control and VCP mutant astrocytes. Data are from two replicates for each sample and show mean ± SD. (**D**) Violin plot showing Δ IR ratio across differential intron retention events in nuclear (red, *n* = 5138) and cytoplasmic (blue, *n* = 2915) fractions. One-sample wilcoxon test adjusted *P* = 1.94e-47 and *P* < 2.00e-145 respectively, **** represents *P* value < 0.0001). Comparing cytoplasmic and nuclear groups with the wilcoxon test showed significantly greater decreases in IR (VCP versus control) in cytoplasmic than nuclear fraction (*P* < 2.2 × 10^−16^). (**E**) Violin plot showing Δ IR ratio (cytoplasmic minus nuclear fractions) across differential IR events in VCP (blue, *n* = 31 358) and CTRL (red, *n* = 28 447). One-sample wilcoxon tests *P* < 2.00e-145 for both. Comparing VCP and CTRL groups showed no differences in nuclear confinement (wilcoxon test *P* = 0.66). (**F**) Nonsense mediated decay (NMD) components (*n* = 120) gene set enrichment analysis of log_2_ fold changes (LFC) in gene expression (VCP versus CTRL) in the cytoplasmic fraction. One-sample wilcoxon test *P* = 2.36 × 10^−7^. (**G**) Violin plots showing log_2_ fold changes in transcript isoform expression in VCP versus CTRL for transcripts annotated in Ensembl as nonsense_mediated_decay (NMD tx) and protein_coding (non-NMD tx) biotypes in cytoplasmic (left, NMD tx *n* = 16 370, non-NMD tx *n* = 91 880, wilcoxon *P* < 2.2 × 10^−16^) and nuclear fractions (right, NMD tx *n* = 16 370, non-NMD tx *n* = 91 880, wilcoxon *P* = 0.11). (**H**) Violin plot showing log2 fold change (VCP versus control) in cytoplasmic transcript abundance for upregulated (blue, *n* = 1033) and downregulated (red, *n* = 1882) nuclear IR events (VCP minus control). Student’s *t*-test was used to compare IR up with IR down. (**I**) Scatterplot of the nuclear Δ IR ratio (VCP minus control) against log2 fold change (LFC) in cytoplasmic spliced transcript abundance in VCP versus control. Black dashed line indicates linear regression correlation (Pearson correlation *R* = −0.21). Reactivity related genes with nuclear IR down and cytoplasmic expression up are labelled. (**J**) Bar graph showing curated overrepresented functional categories (FDR < 0.05) by gene ontology analysis of genes with reduced nuclear IR and increased cytoplasmic mRNA abundance, in VCP versus control. See Supplementary Figure S7G for GO terms in other IR and expression categories. (**K**) Violin plot showing log fold change (VCP versus control) in cytoplasmic mass spectrometry (protein) intensities for upregulated (blue, *n* = 621) and downregulated (red, *n* = 413) nuclear IR events (VCP minus control). Student’s *t*-test was used to compare IR up with IR down (*P* = 0.14). (**L**) Scatterplot of the nuclear Δ IR ratio (nuclear VCP minus nuclear control, *x*-axis) against log fold change (LFC) in mass spectrometry (*y*-axis) in VCP versus control cytoplasmic fractions. Black dashed line indicates linear regression correlation (Pearson correlation, *R* = −0.06). Reactivity related genes with nuclear IR down and cytoplasmic protein up are labelled. (**M**) Bar graph showing curated overrepresented functional categories (FDR < 0.05) by gene ontology analysis of genes with reduced nuclear IR and increased cytoplasmic protein abundance, in VCP versus control. See Supplementary Figure S7H for GO terms in other IR and protein categories. (**N**) Heatmap of astrocyte reactivity markers from Liddelow *et al.*, 2017. Nuclear intron retention (IR), cytoplasmic gene expression (Cyto mRNA) and cytoplasmic mass spectrometry (Cyto protein) data represent the log fold change in VCP versus control.

To gain further mechanistic insight into reactive transformation of astrocytes, we examined the nuclear and cytoplasmic transcriptomes and proteomes in *VCP mutant* astrocytes. To achieve this we performed nuclear–cytoplasmic fractionation of astrocytes, followed by high depth poly(A)+ selected RNA sequencing and mass spectrometry (Figure 4A). Fractionation quality was confirmed by determining that transcripts known to localize to the nucleus (histone *H3*, intronic *GAPDH*, *NEAT*, *MALAT1*) or cytoplasm (exonic *GAPDH*, *Tubulin*) were enriched in the expected compartment, using western blot (Figure 4B), RT-qPCR (Figure 4C), RNA sequencing (Supplementary Figure S6A) and mass spectrometry (Supplementary Figure S6B) of both *VCP* mutant and control astrocytes (40,82). Principal component analysis and unsupervised hierarchical clustering demonstrated that samples segregated based on both fraction (nuclear or cytoplasmic) and genotype (Supplementary Figure S6C and D).

To determine the subcellular location of intron-retaining transcripts, IR focused analysis was performed on nuclear and cytoplasmic isolates. This revealed 5.8-fold more retained introns in nuclear than cytoplasmic fractions (41,138 versus 7,036, respectively, Supplementary Figure S7C and D), consistent with previous reports (38,74,82). Of the 16,317 retained introns identified in whole astrocytes, 92% (*n* = 14,952) were observed in nuclear isolates, 40% (*n* = 6,493) in cytoplasmic isolates, while 37% (*n* = 6,059) were observed in both (Supplementary Figure S7E). Retained introns in both nuclear and cytoplasmic fractions exhibited similar characteristics as in whole astrocytes (shorter in length, higher in GC content, conservation score, RBP binding affinity and lower in splice site strength; Supplementary Figure S8A–F).

Although we observed a reduction in the absolute numbers of retained introns in *VCP* mutant relative to control in both the nucleus (7% less, 34,464 versus 36,943, respectively) and the cytoplasm, this reduction was substantially greater in the cytoplasm (58% less, 2,791 versus 6,607). Comparing IR levels between *VCP* and control, revealed 1.8-fold more significantly differentially retained introns in the cytoplasm (5,138 in 3,065 genes; Supplementary Figure S7B) than the nucleus (2,915 in 2,079 genes; Supplementary Figure S7A). These were decreased in *VCP* mutants in both nuclear (1882/2915, 65%) and cytoplasmic fractions (5089/5138, 99%; Figure 4D, Supplementary Figure S9). Comparing this decrease between the cytoplasm and nucleus confirmed that the cytoplasmic decrease was significantly greater (wilcoxon test, *P* < 2.22 × 10^−16^). This excessive cytoplasmic decrease raises the possibility that IR transcripts in *VCP* mutant astrocytes are subject to either (i) increased nuclear confinement or (ii) enhanced cytoplasmic degradation.

To establish whether differentially retained introns in *VCP* mutant astrocytes differed between the nucleus and cytoplasm, we next examined their IR characteristics. Although similar patterns were noted for GC content, we found that nuclear retained introns that were decreased in *VCP* mutants were shorter, higher in conservation and weaker in RBP binding, compared to introns gained in *VCP* (Supplementary Figure S8G–L). Conversely, cytoplasmic retained introns decreased in *VCP* mutants were longer, lower in conservation and stronger in RBP binding. These opposing nuclear and cytoplasmic IR transcript patterns between *VCP* and control astrocytes support either altered nuclear-to-cytoplasmic transport or differential stability.

To ascertain the degree to which IR transcripts are nuclear confined in *VCP* mutant astrocytes, we compared IR between cytoplasmic and nuclear fractions. Although we noted 10% more differentially retained introns in *VCP* (31,358 events in 9,630 genes) than control (28,447 events in 9,459 genes), the proportion of these that were increased in the nucleus (i.e. nuclear confined) was 99.9% in both conditions (control: 28 410/28 447; *VCP*: 31 330/31 358). Comparing this nuclear confinement between *VCP* and control showed no significant difference (wilcoxon test, *P* = 0.66; Figure 4E). This striking but similar nuclear confinement in both control and *VCP* indicates that astrocytes use IR coupled with nuclear confinement as a strategy to regulate gene expression, consistent with reports in other cell types (30,82).

However, this still leaves unresolved the excessive cytoplasmic decrease in IR within *VCP* mutant astrocytes. To begin to address this, we next examined NMD to establish whether cytoplasmic degradation is responsible for the disproportionate loss of IR. Gene set enrichment analysis of the NMD gene set (*n* = 120) in the cytoplasmic fraction revealed significant upregulation in *VCP* (normalized enrichment score 1.15, enrichment *P* = 0.049), with 94/120 (78%) of the NMD genes being increased (Figure 4F; one-sample wilcoxon test *P* = 2.36 × 10^−7^). By performing differential spliced transcript-level isoform expression analysis in *VCP* versus control fractions and comparing transcripts annotated as NMD substrates with those annotated as protein coding biotypes, we found that cytoplasmic NMD substrate transcripts exhibited significantly lower expression than protein coding biotypes in *VCP* mutant cytoplasm (wilcoxon *P* < 2.2 × 10^−16^). Comparatively there was no significant difference between differential transcript expression between NMD and protein coding biotypes in the nuclear fraction (*P* = 0.11; Figure 4G). This contrast between nuclear and cytoplasmic differential transcript expression according to NMD transcript annotation supports enhanced cytoplasmic NMD activity in *VCP* mutant astrocytes. Taken together, this suggests that increased cytoplasmic NMD activity underlies the excessive cytoplasmic degradation of IR transcripts in *VCP* mutant astrocytes.

To explore whether the nuclear confinement of IR transcripts determines cytoplasmic expression, we next mapped nuclear IR to cytoplasmic spliced transcript abundance. Genes with decreased nuclear IR in *VCP* mutant astrocytes displayed higher cytoplasmic spliced transcript abundance than genes with increased IR (*P* = 1.71 × 10^−35^, *R* = −0.21; Figure 4H and I), suggesting that spliced transcripts are more likely to be exported from the nucleus. Genes with reduced nuclear IR and increased cytoplasmic spliced transcript abundance were over-represented in astrocyte reactivity processes: cell adhesion (*MCAM, COL1A1, ILK*), translation (*EIF2B4, EIF5, EIF3A*) and immune activation (*HLA-B, HLA-C, TGFB1*; Figure 4J). NMD was also enriched, indicating NMD component transcripts themselves are subject to enhanced splicing and cytoplasmic translocation. Of the astrocyte reactivity genes identified in Liddelow *et al.* (24) with a reliable IR event, we observed decreased nuclear IR and increased cytoplasmic expression in 11/30 markers (37%, overlap *P* = 0.002; Figure 4N).

Using mass spectrometry, we detected 1160 proteins in the cytoplasmic fractions of which 580 were increased in protein levels in *VCP* versus control (Supplementary Table S6). Of these 580 proteins, 500 had a reliable IR event, of which 299 (60%) were decreased in nuclear IR and of these, the majority (155/299, 52%) are involved with reactive transformation. Across the entire cytoplasmic proteome, we found no significant difference in protein abundance between those whose cognate transcripts exhibited decreased compared with increased nuclear IR in *VCP* versus control astrocytes (wilcoxon test *P* = 0.46; Figure 4K). However, correlating changes in nuclear IR with changes in cytoplasmic protein abundance revealed a negative correlation (*R* = −0.06; Figure 4L), indicating that decreased nuclear IR is associated with increased cytoplasmic protein. Genes with reduced nuclear IR and increased cytoplasmic protein abundance were over-represented in the infection response, cell adhesion, RNA processing as well as NMD, indicating that an increase in protein levels is a specific phenomenon amongst astrocyte reactivity processes (Figure 4M). Seven of the Liddelow astrocyte reactivity genes were detected in cytoplasmic mass spectrometry of which HSPB1 and HLA-E were both increased in *VCP* mutants, which were also increased in cytoplasmic mRNA (Figure 4N). Comparing cytoplasmic mRNA and protein abundance changes in *VCP* versus control revealed significantly increased mRNA fold changes than that of protein (wilcoxon test *P* = 3.74 × 10^−4^; Supplementary Figure S7F), implicating enhanced cytoplasmic mRNA decay within *VCP* mutant astrocytes. These findings together suggest that under normal circumstances astrocytes employ IR to achieve nuclear confinement of ‘poised’ reactivity transcripts; however in *VCP* mutant astrocytes increased splicing releases them to the cytoplasm permitting their translation (depicted in graphical abstract).

## DISCUSSION

Recent advances in astrocyte biology have implicated reactive astrocytes in ALS pathogenesis (14,22–23,29,81,83). However, despite this accumulating evidence (5–7) and the increased availability of transcriptomic data (8,27,66), the molecular determinants of astrocyte gene expression changes that drive reactive transformation have remained unknown. Although previous transcriptome-wide analyses exposed the reactive nature of ALS astrocytes, they mostly relied on differential gene expression rather than alternative splicing methods (8–9,23,29,66). Combining expression with splicing analyses of the astrocyte transcriptome, coupled with translatome and proteome data, we find that IR is prevalent in healthy quiescent hiPSC-derived astrocytes but that ALS astrocytes share a common decreased IR signature, which is a conserved phenomenon across *VCP, C9orf72* and *SOD1* mutations. This is reproduced in publicly available RNA sequencing datasets in both (i) astrocytes stimulated with TNFα, IL-1α and C1q to undergo reactive transformation (a modest 53% decrease albeit statistically significant) and (ii) astrocytes depleted in TDP-43 *in vivo* (more substantial 83% decrease). Transcripts with decreased IR in ALS and reactive astrocytes displayed increased expression and were over-represented in astrocyte reactivity pathways, supporting IR as a post-transcriptional repressor of reactive transformation (30–32). Furthermore, by re-examining public translational profiling of *SOD1^G37R^* mouse spinal cord astrocytes (67), we observed increased translation of astrocyte reactivity genes that exhibited decreased IR in ALS astrocytes (26). Our study provides insight into the molecular factors regulating reactive transformation of ALS astrocytes, whereby decreased IR might serve to post-transcriptionally enhance astrocyte reactivity genes.

These findings differ substantially from what we and others have established in ALS motor neurons carrying diverse mutations, which exhibit increased IR (41–42,44,46,84–86). Although motor neurons and astrocytes are derived from the same neural precursor cell, they evidently show striking differences in IR regulation during terminal differentiation. Instead, the association between decreased IR and astrocyte reactivity resembles other immune cells, such as lymphocytes and macrophages, where there is an inverse relationship between global IR levels and the cellular activation state (31–34,47). We speculate that the opposing shift in IR between motor neurons and astrocytes is a consequence of distinct cell-type responses to ALS pathogenic processes, such as TDP-43 proteinopathy (71), extrinsic stressors (87), neuronal injury (8,88) or even reactive transformation itself. Although *VCP, C9orf72* and *SOD1* mutations are pathologically distinct with divergent effects, a common theme is that they are each linked to perturbed RNA processing and/or RBP mislocalization, which may be responsible for these ALS cell-type specific alterations in IR (3,41). In support of this, TDP-43 mislocalization triggers neurons to upregulate immune response pathways (NF-kB and type 1 interferon) via the cGAS-STING pathway (89), whereas TDP-43 proteinopathy in astrocytes has been shown to be associated with metabolic dysregulation via cAMP and calcium signalling (90). These divergent responses of astrocytes and motor neurons may differentially affect key splicing factors, provoking deregulation of the spliceosome complex and altering its finely-tuned ability to recognize and act on splicing signals (91–93). Future functional studies with cell-type specific targeted disruption of spliceosome components, as well as splicing enhancers and repressors, would help to elucidate the mechanisms of this differential IR shift and whether it directly contributes to disease pathogenesis (94).

It has been proposed that intron-containing mRNAs may function to encourage their nuclear confinement, preventing nuclear leak and translation (74,82). Thus, an important question is whether transcripts exhibiting decreased IR in ALS astrocytes are able to escape nuclear confinement and undergo nuclear-export. To address this, we used nuclear-cytoplasmic fractionation and found decreased IR in both nuclear and cytoplasmic compartments in *VCP* mutant astrocytes. We identified more than double the number of differential IR events (*VCP* mutant versus control) in the cytoplasm (5,138) than the nucleus (1,882) and although there was an overall decrease in IR in both *VCP* fractions, the proportion lost was dramatically greater in the cytoplasm (99% versus 65%). Although there was no change in the strength of nuclear confinement of IR transcripts, there was a substantial loss of transcripts annotated as NMD substrates from the cytoplasm in *VCP* mutant astrocytes. We further established that NMD components exhibited decreased nuclear IR and increased cytoplasmic abundance in *VCP* mutant astrocytes. This suggests that, together with astrocyte reactivity transcripts, NMD components are subject to enhanced splicing enabling their cytoplasmic translocation. While it is possible that other mechanisms regulating IR transcripts in the cytoplasm are responsible, such as targeted subcellular localization, RBP sequestering or cytoplasmic splicing, the most likely explanation for the excessive cytoplasmic degradation of IR transcripts in *VCP* mutant astrocytes is enhanced NMD activity (42,95–96). This raises the possibility that NMD itself is a key part of reactive transformation, which would be consistent with studies reporting that IR-NMD coupling has additional roles in regulating the stress response, beyond its well established post-transcriptional quality control mechanism serving to degrade PTC-containing IR transcripts (35–36,74,97–101). Indeed, perturbed IR-NMD crosstalk has been shown in neuroinflammation and ALS, where it may act to liberate bound RBPs for further splicing modulation (32,102–105).

Consistent with enhanced NMD in *VCP* mutant astrocytes, we also found that nuclear transcripts with decreased IR manifest higher cytoplasmic spliced transcript abundance than nuclear transcripts with increased IR. We observed a major cluster of astrocyte reactivity related genes in *VCP* mutant astrocytes that are subject to loss of nuclear IR and increased cytoplasmic spliced transcript abundance, including many of the established astrocyte reactivity genes identified by Liddelow *et al.* (24). Interestingly, of these we found multiple HLA class 1 genes, which when overexpressed protect motor neurons from reactive astrocyte toxicity (106). Within this cluster we also identified enrichment of other genes involved with the extracellular-matrix (e.g. *COL1A1, MMP24, ADAM19*), focal adhesion (e.g. *MCAM, ILK, EPHA2, ITGB1*) and immune-activation (e.g. *TGFB1, NFKB1, NFKB2, CD68, IL32*), consistent with processes previously identified in ALS astrocytes (8,15–16,29,66). This indicates that transcripts regulating multiple facets of astrocyte reactivity exhibit enhanced nuclear splicing in *VCP* mutant astrocytes that may allow nuclear-export to the cytoplasm for translation, facilitating reactive transformation.

Overall, our study provides new insights into the coordinated role of IR coupled with gene expression in astrocytes and its aberration in ALS. Astrocytes employ IR to post-transcriptionally repress reactivity genes; however, ALS astrocytes undergo augmented splicing and lose this homeostatic regulation, which may be an initial compensatory mechanism that may become maladaptive over time. Further investigation into the impact of this phenomenon on neighbouring neurons will enable an improved understanding of ALS, as well as other neurodegenerative diseases characterized by aberrant astrocyte reactivity. Our study raises the prospect of therapeutically targeting astrocyte reactivity through reinstating the physiological IR programme by manipulation of the splicing process, for example using antisense oligonucleotides.

## DATA AVAILABILITY

All raw and processed mRNA sequencing data generated in this study have been deposited in the NCBI Sequence Read Archive (BioProject Gene Expression Omnibus) under accession number GSE160133. RAW Mass Spectrometry data have been deposited to the ProteomeXchange Consortium (http://proteomecentral.proteomexchange.org) via the PRIDE partner repository with the dataset identifier PXD022604. Code is available through GitHub https://github.com/ojziff/ALS_astrocyte_intron_retention. hiPSC-derived astrocytes carrying ALS mutations are available at GSE142730 (C9orf72), GSE102902 and GSE99843 (SOD1 mutants and control respectively). Cytokine-stimulated hiPSC-derived astrocytes are available at syn21861181. *TARDBP* knockout mouse spinal cord astrocyte specific RNA-seq is available at GSE156542. Mouse SOD1 astrocyte TRAP-seq is available at GSE74724.

## Supplementary Material

gkab115_Supplemental_Files

## References

[1] Neumann M. , Sampathu D.M., Kwong L.K., Truax A.C., Micsenyi M.C., Chou T.T., Bruce J., Schuck T., Grossman M., Clark C.M. et al. Ubiquitinated TDP-43 in frontotemporal lobar degeneration and amyotrophic lateral sclerosis. Science. 2006; 314:130–133. 17023659 10.1126/science.1134108

[2] Kiernan M.C. , Vucic S., Talbot K., McDermott C.J., Hardiman O., Shefner J.M., Al-Chalabi A., Huynh W., Cudkowicz M., Talman P. et al. Improving clinical trial outcomes in amyotrophic lateral sclerosis. Nat. Rev. Neurol. 2020; 17:104–118. 33340024 10.1038/s41582-020-00434-z PMC7747476

[3] Shatunov A. , Al-Chalabi A. The genetic architecture of ALS. Neurobiol. Dis. 2020; 147:105156. 33130222 10.1016/j.nbd.2020.105156

[4] Johnson J.O. , Mandrioli J., Benatar M., Abramzon Y., Van Deerlin V.M., Trojanowski J.Q., Gibbs J.R., Brunetti M., Gronka S., Wuu J. et al. Exome sequencing reveals VCP mutations as a cause of familial ALS. Neuron. 2010; 68:857–864. 21145000 10.1016/j.neuron.2010.11.036 PMC3032425

[5] Serio A. , Patani R. Concise Review: The cellular conspiracy of amyotrophic lateral sclerosis. Stem Cells. 2018; 36:293–303. 29235200 10.1002/stem.2758

[6] Tyzack G. , Lakatos A., Patani R. Human stem cell-derived Astrocytes: Specification and relevance for neurological disorders. Curr. Stem Cell. Rep. 2016; 2:236–247. 27547709 10.1007/s40778-016-0049-1 PMC4972864

[7] Sofroniew M.V. , Vinters H.V. Astrocytes: biology and pathology. Acta Neuropathol. 2010; 119:7–35. 20012068 10.1007/s00401-009-0619-8 PMC2799634

[8] Tyzack G.E. , Hall C.E., Sibley C.R., Cymes T., Forostyak S., Carlino G., Meyer I.F., Schiavo G., Zhang S.-C., Gibbons G.M. et al. A neuroprotective astrocyte state is induced by neuronal signal EphB1 but fails in ALS models. Nat. Commun. 2017; 8:1164. 29079839 10.1038/s41467-017-01283-z PMC5660125

[9] Zhao C. , Devlin A.-C., Chouhan A.K., Selvaraj B.T., Stavrou M., Burr K., Brivio V., He X., Mehta A.R., Story D. et al. Mutant C9orf72 human iPSC-derived astrocytes cause non-cell autonomous motor neuron pathophysiology. Glia. 2019; 68:1046–1106. 31841614 10.1002/glia.23761 PMC7078830

[10] Serio A. , Bilican B., Barmada S.J., Ando D.M., Zhao C., Siller R., Burr K., Haghi G., Story D., Nishimura A.L. et al. Astrocyte pathology and the absence of non-cell autonomy in an induced pluripotent stem cell model of TDP-43 proteinopathy. Proc. Natl. Acad. Sci. USA. 2013; 110:4697–4702. 23401527 10.1073/pnas.1300398110 PMC3607024

[11] Hall C.E. , Yao Z., Choi M., Tyzack G.E., Serio A., Luisier R., Harley J., Preza E., Arber C., Crisp S.J. et al. Progressive Motor Neuron Pathology and the Role of Astrocytes in a Human Stem Cell Model of VCP-Related ALS. Cell Rep. 2017; 19:1739–1749. 28564594 10.1016/j.celrep.2017.05.024 PMC5464993

[12] Di Giorgio F.P. , Carrasco M.A., Siao M.C., Maniatis T., Eggan K. Non-cell autonomous effect of glia on motor neurons in an embryonic stem cell-based ALS model. Nat. Neurosci. 2007; 10:608–614. 17435754 10.1038/nn1885 PMC3139463

[13] Meyer K. , Ferraiuolo L., Miranda C.J., Likhite S., McElroy S., Renusch S., Ditsworth D., Lagier-Tourenne C., Smith R.A., Ravits J. et al. Direct conversion of patient fibroblasts demonstrates non-cell autonomous toxicity of astrocytes to motor neurons in familial and sporadic ALS. Proc. Natl. Acad. Sci. USA. 2014; 111:829–832. 24379375 10.1073/pnas.1314085111 PMC3896192

[14] Yamanaka K. , Chun S.J., Boillee S., Fujimori-Tonou N., Yamashita H., Gutmann D.H., Takahashi R., Misawa H., Cleveland D.W. Astrocytes as determinants of disease progression in inherited amyotrophic lateral sclerosis. Nat. Neurosci. 2008; 11:251–253. 18246065 10.1038/nn2047 PMC3137510

[15] Nagai M. , Re D.B., Nagata T., Chalazonitis A., Jessell T.M., Wichterle H., Przedborski S. Astrocytes expressing ALS-linked mutated SOD1 release factors selectively toxic to motor neurons. Nat. Neurosci. 2007; 10:615–622. 17435755 10.1038/nn1876 PMC3799799

[16] Haidet-Phillips A.M. , Hester M.E., Miranda C.J., Meyer K., Braun L., Frakes A., Song S., Likhite S., Murtha M.J., Foust K.D. et al. Astrocytes from familial and sporadic ALS patients are toxic to motor neurons. Nat. Biotechnol. 2011; 29:824–828. 21832997 10.1038/nbt.1957 PMC3170425

[17] Marchetto M.C.N. , Muotri A.R., Mu Y., Smith A.M., Cezar G.G., Gage F.H Non-cell-autonomous effect of human SOD1 G37R astrocytes on motor neurons derived from human embryonic stem cells. Cell Stem Cell. 2008; 3:649–657. 19041781 10.1016/j.stem.2008.10.001

[18] Brohawn D.G. , O’Brien L.C., Bennett J.P. Jr RNAseq Analyses Identify Tumor Necrosis Factor-Mediated Inflammation as a Major Abnormality in ALS Spinal Cord. PLoS One. 2016; 11:e0160520. 27487029 10.1371/journal.pone.0160520 PMC4972368

[19] Cooper-Knock J. , Green C., Altschuler G., Wei W., Bury J.J., Heath P.R., Wyles M., Gelsthorpe C., Highley J.R., Lorente-Pons A. et al. A data-driven approach links microglia to pathology and prognosis in amyotrophic lateral sclerosis. Acta Neuropathol. Commun. 2017; 5:23. 28302159 10.1186/s40478-017-0424-x PMC5353945

[20] Tam O.H. , Rozhkov N.V., Shaw R., Kim D., Hubbard I., Fennessey S., Propp N. NYGC ALS Consortium NYGC ALS Consortium Fagegaltier D., Harris B.T. et al. Postmortem cortex samples identify distinct molecular subtypes of ALS: Retrotransposon activation, oxidative stress, and activated glia. Cell Rep. 2019; 29:1164–1177. 31665631 10.1016/j.celrep.2019.09.066 PMC6866666

[21] D’Erchia A.M. , Gallo A., Manzari C., Raho S., Horner D.S., Chiara M., Valletti A., Aiello I., Mastropasqua F., Ciaccia L. et al. Massive transcriptome sequencing of human spinal cord tissues provides new insights into motor neuron degeneration in ALS. Sci. Rep. 2017; 7:10046. 28855684 10.1038/s41598-017-10488-7 PMC5577269

[22] Schiffer D. , Cordera S., Cavalla P., Migheli A. Reactive astrogliosis of the spinal cord in amyotrophic lateral sclerosis. J. Neurol. Sci. 1996; 139:27–33. 8899654 10.1016/0022-510x(96)00073-1

[23] Nagy D. , Kato T., Kushner P.D. Reactive astrocytes are widespread in the cortical gray matter of amyotrophic lateral sclerosis. J. Neurosci. Res. 1994; 38:336–347. 7523689 10.1002/jnr.490380312

[24] Liddelow S.A. , Guttenplan K.A., Clarke L.E., Bennett F.C., Bohlen C.J., Schirmer L., Bennett M.L., Münch A.E., Chung W.-S., Peterson T.C. et al. Neurotoxic reactive astrocytes are induced by activated microglia. Nature. 2017; 541:481–487. 28099414 10.1038/nature21029 PMC5404890

[25] Cahoy J.D. , Emery B., Kaushal A., Foo L.C., Zamanian J.L., Christopherson K.S., Xing Y., Lubischer J.L., Krieg P.A., Krupenko S.A. et al. A transcriptome database for astrocytes, neurons, and oligodendrocytes: a new resource for understanding brain development and function. J. Neurosci. 2008; 28:264–278. 18171944 10.1523/JNEUROSCI.4178-07.2008 PMC6671143

[26] Zamanian J.L. , Xu L., Foo L.C., Nouri N., Zhou L., Giffard R.G., Barres B.A. Genomic analysis of reactive astrogliosis. J. Neurosci. 2012; 32:6391–6410. 22553043 10.1523/JNEUROSCI.6221-11.2012 PMC3480225

[27] Barbar L. , Jain T., Zimmer M., Kruglikov I., Sadick J.S., Wang M., Kalpana K., Rose I.V.L., Burstein S.R., Rusielewicz T. et al. CD49f is a novel marker of functional and reactive human iPSC-Derived astrocytes. Neuron. 2020; 107:436–453. 32485136 10.1016/j.neuron.2020.05.014 PMC8274549

[28] Zhang Y. , Chen K., Sloan S.A., Bennett M.L., Scholze A.R., O’Keeffe S., Phatnani H.P., Guarnieri P., Caneda C., Ruderisch N. et al. An RNA-sequencing transcriptome and splicing database of glia, neurons, and vascular cells of the cerebral cortex. J. Neurosci. 2014; 34:11929–11947. 25186741 10.1523/JNEUROSCI.1860-14.2014 PMC4152602

[29] Vargas M.R. , Pehar M., Díaz-Amarilla P.J., Beckman J.S., Barbeito L. Transcriptional profile of primary astrocytes expressing ALS-linked mutant SOD1. J. Neurosci. Res. 2008; 86:3515–3525. 18683239 10.1002/jnr.21797 PMC4048747

[30] Mauger O. , Lemoine F., Scheiffele P. Targeted intron retention and excision for rapid gene regulation in response to neuronal activity. Neuron. 2016; 92:1266–1278. 28009274 10.1016/j.neuron.2016.11.032

[31] Naro C. , Jolly A., Di Persio S., Bielli P., Setterblad N., Alberdi A.J., Vicini E., Geremia R., De la Grange P., Sette C. An orchestrated intron retention program in meiosis controls timely usage of transcripts during germ cell differentiation. Dev. Cell. 2017; 41:82–93. 28366282 10.1016/j.devcel.2017.03.003 PMC5392497

[32] Yap K. , Lim Z.Q., Khandelia P., Friedman B., Makeyev E.V. Coordinated regulation of neuronal mRNA steady-state levels through developmentally controlled intron retention. Genes Dev. 2012; 26:1209–1223. 22661231 10.1101/gad.188037.112 PMC3371409

[33] Green I.D. , Pinello N., Song R., Lee Q., Halstead J.M., Kwok C.-T., Wong A.C.H., Nair S.S., Clark S.J., Roediger B. et al. Macrophage development and activation involve coordinated intron retention in key inflammatory regulators. Nucleic Acids Res. 2020; 48:6513–6529. 32449925 10.1093/nar/gkaa435 PMC7337907

[34] Ni T. , Yang W., Han M., Zhang Y., Shen T., Nie H., Zhou Z., Dai Y., Yang Y., Liu P. et al. Global intron retention mediated gene regulation during CD4+ T cell activation. Nucleic Acids Res. 2016; 44:6817–6829. 27369383 10.1093/nar/gkw591 PMC5001615

[35] Wong J.J.-L. , Au A.Y.M., Ritchie W., Rasko J.E.J. Intron retention in mRNA: No longer nonsense: Known and putative roles of intron retention in normal and disease biology. BioEssays. 2016; 38:41–49. 26612485 10.1002/bies.201500117

[36] Wong J.J.-L. , Ritchie W., Ebner O.A., Selbach M., Wong J.W.H., Huang Y., Gao D., Pinello N., Gonzalez M., Baidya K. et al. Orchestrated intron retention regulates normal granulocyte differentiation. Cell. 2013; 154:583–595. 23911323 10.1016/j.cell.2013.06.052

[37] Jaillon O. , Bouhouche K., Gout J.-F., Aury J.-M., Noel B., Saudemont B., Nowacki M., Serrano V., Porcel B.M., Ségurens B. et al. Translational control of intron splicing in eukaryotes. Nature. 2008; 451:359–362. 18202663 10.1038/nature06495

[38] Boutz P.L. , Bhutkar A., Sharp P.A. Detained introns are a novel, widespread class of post-transcriptionally spliced introns. Genes Dev. 2015; 29:63–80. 25561496 10.1101/gad.247361.114 PMC4281565

[39] Lemieux C. , Marguerat S., Lafontaine J., Barbezier N., Bähler J., Bachand F. A Pre-mRNA degradation pathway that selectively targets intron-containing genes requires the nuclear poly(A)-binding protein. Mol. Cell. 2011; 44:108–119. 21981922 10.1016/j.molcel.2011.06.035

[40] Bahar Halpern K. , Caspi I., Lemze D., Levy M., Landen S., Elinav E., Ulitsky I., Itzkovitz S. Nuclear Retention of mRNA in Mammalian Tissues. Cell Rep. 2015; 13:2653–2662. 26711333 10.1016/j.celrep.2015.11.036 PMC4700052

[41] Luisier R. , Tyzack G.E., Hall C.E., Mitchell J.S., Devine H., Taha D.M., Malik B., Meyer I., Greensmith L., Newcombe J. et al. Intron retention and nuclear loss of SFPQ are molecular hallmarks of ALS. Nat. Commun. 2018; 9:2010. 29789581 10.1038/s41467-018-04373-8 PMC5964114

[42] Tyzack G.E. , Neeves J., Klein P., Crerar H., Ziff O., Taha D.M., Luisier R., Luscombe N.M., Patani R. An aberrant cytoplasmic intron retention program is a blueprint RBP mislocalization in VCP-related ALS. 2021; BioRxiv doi:21 July 2020, preprint: not peer reviewed 10.1101/2020.07.20.211557. PMC8370440 33693641

[43] Hsieh Y.-C. , Guo C., Yalamanchili H.K., Abreha M., Al-Ouran R., Li Y., Dammer E.B., Lah J.J., Levey A.I., Bennett D.A. et al. Tau-Mediated disruption of the spliceosome triggers cryptic RNA splicing and neurodegeneration in Alzheimer's disease. Cell Rep. 2019; 29:301–316. 31597093 10.1016/j.celrep.2019.08.104 PMC6919331

[44] Humphrey J. , Birsa N., Milioto C., McLaughlin M., Ule A.M., Robaldo D., Eberle A.B., Kräuchi R., Bentham M., Brown A.-L. et al. FUS ALS-causative mutations impair FUS autoregulation and splicing factor networks through intron retention. Nucleic Acids Res. 2020; 48:6889–6905. 32479602 10.1093/nar/gkaa410 PMC7337901

[45] Adusumalli S. , Ngian Z.-K., Lin W.-Q., Benoukraf T., Ong C.-T. Increased intron retention is a post-transcriptional signature associated with progressive aging and Alzheimer’s disease. Aging Cell. 2019; 18:e12928. 30868713 10.1111/acel.12928 PMC6516162

[46] Wang Q. , Conlon E., Manley J., Rio D. Widespread intron retention impairs protein homeostasis in C9orf72 ALS brains. Genome Res. 2020; 30:1705–1715. 33055097 10.1101/gr.265298.120 PMC7706729

[47] Ullrich S. , Guigó R. Dynamic changes in intron retention are tightly associated with regulation of splicing factors and proliferative activity during B-cell development. Nucleic Acids Res. 2020; 48:1327–1340. 31879760 10.1093/nar/gkz1180 PMC7026658

[48] Kelley K.W. , Ben Haim L., Schirmer L., Tyzack G.E., Tolman M., Miller J.G., Tsai H.-H., Chang S.M., Molofsky A.V., Yang Y. et al. Kir4.1-dependent astrocyte-fast motor neuron interactions are required for peak strength. Neuron. 2018; 98:306–319. 29606582 10.1016/j.neuron.2018.03.010 PMC5919779

[49] Thelin E.P. , Hall C.E., Tyzack G.E., Frostell A., Giorgi-Coll S., Alam A., Carpenter K.L.H., Mitchell J., Tajsic T., Hutchinson P.J. et al. Delineating astrocytic cytokine responses in a human stem cell model of neural trauma. J. Neurotrauma. 2020; 37:93–105. 31452443 10.1089/neu.2019.6480 PMC6921298

[50] Clarke B.E. , Taha D.M., Ziff O.J., Alam A., Thelin E.P., García N.M., Helmy A., Patani R. Human stem cell-derived astrocytes exhibit region-specific heterogeneity in their secretory profiles. Brain. 2020; 143:e85. 32895697 10.1093/brain/awaa258 PMC7586081

[51] Ziff O.J. , Patani R. Harnessing cellular aging in human stem cell models of amyotrophic lateral sclerosis. Aging Cell. 2019; 18:e12862. 30565851 10.1111/acel.12862 PMC6351881

[52] Dobin A. , Davis C.A., Schlesinger F., Drenkow J., Zaleski C., Jha S., Batut P., Chaisson M., Gingeras T.R. STAR: ultrafast universal RNA-seq aligner. Bioinformatics. 2013; 29:15–21. 23104886 10.1093/bioinformatics/bts635 PMC3530905

[53] Anders S. , Pyl P.T., Huber W. HTSeq—a Python framework to work with high-throughput sequencing data. Bioinformatics. 2014; 31:166–169. 25260700 10.1093/bioinformatics/btu638 PMC4287950

[54] Ewels P. , Peltzer A., Fillinger S., Patel H., Alneberg J., Wilm A., Garcia M.U., Tommaso P.D., Nahnsen S. The nf-core framework for community-curated bioinformatics pipelines. Nature Biotech. 2020; 38:276–278. 10.1038/s41587-020-0439-x 32055031

[55] Love M.I. , Huber W., Anders S. Moderated estimation of fold change and dispersion for RNA-seq data with DESeq2. Genome Biol. 2014; 15:550. 25516281 10.1186/s13059-014-0550-8 PMC4302049

[56] Tapial J. , Ha K.C.H., Sterne-Weiler T., Gohr A., Braunschweig U., Hermoso-Pulido A., Quesnel-Vallières M., Permanyer J., Sodaei R., Marquez Y. et al. An atlas of alternative splicing profiles and functional associations reveals new regulatory programs and genes that simultaneously express multiple major isoforms. Genome Res. 2017; 27:1759–1768. 28855263 10.1101/gr.220962.117 PMC5630039

[57] Goldstein L.D. , Cao Y., Pau G., Lawrence M., Wu T.D., Seshagiri S., Gentleman R. Prediction and quantification of splice events from RNA-Seq Data. PLoS One. 2016; 11:e0156132. 27218464 10.1371/journal.pone.0156132 PMC4878813

[58] Middleton R. , Gao D., Thomas A., Singh B., Au A., Wong J.J.-L., Bomane A., Cosson B., Eyras E., Rasko J.E.J. et al. IRFinder: assessing the impact of intron retention on mammalian gene expression. Genome Biol. 2017; 18:51. 28298237 10.1186/s13059-017-1184-4 PMC5353968

[59] Raudvere U. , Kolberg L., Kuzmin I., Arak T., Adler P., Peterson H., Vilo J. g:Profiler: a web server for functional enrichment analysis and conversions of gene lists (2019 update). Nucleic Acids Res. 2019; 47:W191–W198. 31066453 10.1093/nar/gkz369 PMC6602461

[60] Korotkevich G. , Sukhov V., Sergushichev A. Fast Gene Set Enrichment Analysis. 2019; Cold Spring Harbor Laboratory doi:10.1101/060012.

[61] Gene Ontology Consortium Nuclear-transcribed mRNA catabolic process, nonsense-mediated decay. 2021; GO:0000184.

[62] Attig J. , Agostini F., Gooding C., Chakrabarti A.M., Singh A., Haberman N., Zagalak J.A., Emmett W., Smith C.W.J., Luscombe N.M. et al. Heteromeric RNP assembly at LINEs controls lineage-specific RNA processing. Cell. 2018; 174:1067–1081. 30078707 10.1016/j.cell.2018.07.001 PMC6108849

[63] Sloan C.A. , Chan E.T., Davidson J.M., Malladi V.S., Strattan J.S., Hitz B.C., Gabdank I., Narayanan A.K., Ho M., Lee B.T. et al. ENCODE data at the ENCODE portal. Nucleic Acids Res. 2016; 44:D726–D732. 26527727 10.1093/nar/gkv1160 PMC4702836

[64] Yeo G. , Burge C.B. Maximum entropy modeling of short sequence motifs with applications to RNA splicing signals. J. Comput. Biol. 2004; 11:377–394. 15285897 10.1089/1066527041410418

[65] Bray N.L. , Pimentel H., Melsted P., Pachter L. Near-optimal probabilistic RNA-seq quantification. Nat. Biotechnol. 2016; 34:525–527. 27043002 10.1038/nbt.3519

[66] Birger A. , Ben-Dor I., Ottolenghi M., Turetsky T., Gil Y., Sweetat S., Perez L., Belzer V., Casden N., Steiner D. et al. Human iPSC-derived astrocytes from ALS patients with mutated C9ORF72 show increased oxidative stress and neurotoxicity. EBioMedicine. 2019; 50:274–289. 31787569 10.1016/j.ebiom.2019.11.026 PMC6921360

[67] Sun S. , Sun Y., Ling S.-C., Ferraiuolo L., McAlonis-Downes M., Zou Y., Drenner K., Wang Y., Ditsworth D., Tokunaga S. et al. Translational profiling identifies a cascade of damage initiated in motor neurons and spreading to glia in mutant SOD1-mediated ALS. Proc. Natl. Acad. Sci. USA. 2015; 112:E6993–E7002. 26621731 10.1073/pnas.1520639112 PMC4687558

[68] Peng A.Y.T. , Agrawal I., Ho W.Y., Yen Y.-C., Pinter A.J., Liu J., Phua Q.X.C., Koh K.B., Chang J.-C., Sanford E. et al. Loss of TDP-43 in astrocytes leads to motor deficits by triggering A1-like reactive phenotype and triglial dysfunction. Proc. Natl. Acad. Sci. USA. 2020; 117:29101–29112. 33127758 10.1073/pnas.2007806117 PMC7682406

[69] Broseus L. , Ritchie W. Challenges in detecting and quantifying intron retention from next generation sequencing data. Comput. Struct. Biotechnol. J. 2020; 18:501–508. 32206209 10.1016/j.csbj.2020.02.010 PMC7078297

[70] Zhang X. , Smits A.H., van Tilburg G.B., Ovaa H., Huber W., Vermeulen M. Proteome-wide identification of ubiquitin interactions using UbIA-MS. Nat. Protoc. 2018; 13:530–550. 29446774 10.1038/nprot.2017.147

[71] Smethurst P. , Risse E., Tyzack G.E., Mitchell J.S., Taha D.M., Chen Y.-R., Newcombe J., Collinge J., Sidle K., Patani R. Distinct responses of neurons and astrocytes to TDP-43 proteinopathy in amyotrophic lateral sclerosis. Brain. 2020; 143:430–440. 32040555 10.1093/brain/awz419 PMC7009461

[72] Levine J. , Kwon E., Paez P., Yan W., Czerwieniec G., Loo J.A., Sofroniew M.V., Wanner I.-B. Traumatically injured astrocytes release a proteomic signature modulated by STAT3-dependent cell survival. Glia. 2016; 64:668–694. 26683444 10.1002/glia.22953 PMC4805454

[73] Galante P.A.F. , Sakabe N.J., Kirschbaum-Slager N., de Souza S.J Detection and evaluation of intron retention events in the human transcriptome. RNA. 2004; 10:757–765. 15100430 10.1261/rna.5123504 PMC1370565

[74] Braunschweig U. , Barbosa-Morais N.L., Pan Q., Nachman E.N., Alipanahi B., Gonatopoulos-Pournatzis T., Frey B., Irimia M., Blencowe B.J. Widespread intron retention in mammals functionally tunes transcriptomes. Genome Res. 2014; 24:1774–1786. 25258385 10.1101/gr.177790.114 PMC4216919

[75] Van Nostrand E.L. , Freese P., Pratt G.A., Wang X., Wei X., Xiao R., Blue S.M., Chen J.-Y., Cody N.A.L., Dominguez D. et al. A large-scale binding and functional map of human RNA-binding proteins. Nature. 2020; 583:711–719. 32728246 10.1038/s41586-020-2077-3 PMC7410833

[76] Ge Y. , Porse B.T. The functional consequences of intron retention: alternative splicing coupled to NMD as a regulator of gene expression. Bioessays. 2014; 36:236–243. 24352796 10.1002/bies.201300156

[77] Jacob A.G. , Smith C.W.J Intron retention as a component of regulated gene expression programs. Hum. Genet. 2017; 136:1043–1057. 28391524 10.1007/s00439-017-1791-x PMC5602073

[78] Neo S.H. , Tang B.L. Collagen 1 signaling at the central nervous system injury site and astrogliosis. Neural Regener. Res. 2017; 12:1600–1601. 10.4103/1673-5374.217323 PMC5696833 29171417

[79] Teh D.B.L. , Prasad A., Jiang W., Ariffin M.Z., Khanna S., Belorkar A., Wong L., Liu X., All A.H Transcriptome analysis reveals neuroprotective aspects of human reactive astrocytes induced by interleukin 1β. Sci. Rep. 2017; 7:13988. 29070875 10.1038/s41598-017-13174-w PMC5656635

[80] Hara M. , Kobayakawa K., Ohkawa Y., Kumamaru H., Yokota K., Saito T., Kijima K., Yoshizaki S., Harimaya K., Nakashima Y. et al. Interaction of reactive astrocytes with type I collagen induces astrocytic scar formation through the integrin-N-cadherin pathway after spinal cord injury. Nat. Med. 2017; 23:818–828. 28628111 10.1038/nm.4354

[81] Ajmone-Cat M.A. , Onori A., Toselli C., Stronati E., Morlando M., Bozzoni I., Monni E., Kokaia Z., Lupo G., Minghetti L. et al. Increased FUS levels in astrocytes leads to astrocyte and microglia activation and neuronal death. Sci. Rep. 2019; 9:4572. 30872738 10.1038/s41598-019-41040-4 PMC6418113

[82] Price A.J. , Hwang T., Tao R., Burke E.E., Rajpurohit A., Shin J.H., Hyde T.M., Kleinman J.E., Jaffe A.E., Weinberger D.R. Characterizing the nuclear and cytoplasmic transcriptomes in developing and mature human cortex uncovers new insight into psychiatric disease gene regulation. Genome Res. 2020; 30:1–11. 31852722 10.1101/gr.250217.119 PMC6961577

[83] Guttenplan K.A. , Weigel M.K., Adler D.I., Couthouis J., Liddelow S.A., Gitler A.D., Barres B.A. Knockout of reactive astrocyte activating factors slows disease progression in an ALS mouse model. Nat. Commun. 2020; 11:3753. 32719333 10.1038/s41467-020-17514-9 PMC7385161

[84] Sznajder Ł.J. , Thomas J.D., Carrell E.M., Reid T., McFarland K.N., Cleary J.D., Oliveira R., Nutter C.A., Bhatt K., Sobczak K. et al. Intron retention induced by microsatellite expansions as a disease biomarker. Proc. Natl. Acad. Sci. USA. 2018; 115:4234–4239. 29610297 10.1073/pnas.1716617115 PMC5910826

[85] Bai G. , Lipton S.A. Aberrant RNA splicing minireview in sporadic amyotrophic lateral sclerosis. Neuro. 1998; 20:363–366. 10.1016/s0896-6273(00)80979-4 9539113

[86] Flomen R. , Makoff A. Increased RNA editing in EAAT2 pre-mRNA from amyotrophic lateral sclerosis patients: involvement of a cryptic polyadenylation site. Neurosci. Lett. 2011; 497:139–143. 21569822 10.1016/j.neulet.2011.04.047

[87] Hock E.-M. , Maniecka Z., Hruska-Plochan M., Reber S., Laferrière F., Sahadevan M K S., Ederle H., Gittings L., Pelkmans L., Dupuis L. et al. Hypertonic stress causes cytoplasmic translocation of neuronal, but not astrocytic, FUS due to impaired transportin function. Cell Rep. 2018; 24:987–1000. 30044993 10.1016/j.celrep.2018.06.094

[88] Xu Q. , Walker D., Bernardo A., Brodbeck J., Balestra M.E., Huang Y. Intron-3 retention/splicing controls neuronal expression of apolipoprotein E in the CNS. J. Neurosci. 2008; 28:1452–1459. 18256266 10.1523/JNEUROSCI.3253-07.2008 PMC6671590

[89] Yu C.-H. , Davidson S., Harapas C.R., Hilton J.B., Mlodzianoski M.J., Laohamonthonkul P., Louis C., Low R.R.J., Moecking J., De Nardo D. et al. TDP-43 Triggers Mitochondrial DNA Release via mPTP to Activate cGAS/STING in ALS. Cell. 2020; 183:636–649. 33031745 10.1016/j.cell.2020.09.020 PMC7599077

[90] Velebit J. , Horvat A., Smolič T., Prpar Mihevc S., Rogelj B., Zorec R., Vardjan N. Astrocytes with TDP-43 inclusions exhibit reduced noradrenergic cAMP and Ca2+ signaling and dysregulated cell metabolism. Sci. Rep. 2020; 10:6003. 32265469 10.1038/s41598-020-62864-5 PMC7138839

[91] Tsuiji H. , Iguchi Y., Furuya A., Kataoka A., Hatsuta H., Atsuta N., Tanaka F., Hashizume Y., Akatsu H., Murayama S. et al. Spliceosome integrity is defective in the motor neuron diseases ALS and SMA. EMBO Mol. Med. 2013; 5:221–234. 23255347 10.1002/emmm.201202303 PMC3569639

[92] La Cognata V. , Gentile G., Aronica E., Cavallaro S. Splicing players are differently expressed in sporadic amyotrophic lateral sclerosis molecular clusters and brain regions. Cells. 2020; 9:159. 10.3390/cells9010159 PMC7017305 31936368

[93] Fratta P. , Isaacs A.M. The snowball effect of RNA binding protein dysfunction in amyotrophic lateral sclerosis. Brain. 2018; 141:1236–1238. 29701791 10.1093/brain/awy091 PMC5917769

[94] Wang Y. , Patani R. Novel therapeutic targets for amyotrophic lateral sclerosis: ribonucleoproteins and cellular autonomy. Expert Opin. Ther. Targets. 2020; 24:971–984. 32746659 10.1080/14728222.2020.1805734

[95] Buckley P.T. , Khaladkar M., Kim J., Eberwine J. Cytoplasmic intron retention, function, splicing, and the sentinel RNA hypothesis. Wiley Interdiscip. Rev. RNA. 2014; 5:223–230. 24190870 10.1002/wrna.1203 PMC4449146

[96] Buckley P.T. , Lee M.T., Sul J.-Y., Miyashiro K.Y., Bell T.J., Fisher S.A., Kim J., Eberwine J. Cytoplasmic intron sequence-retaining transcripts can be dendritically targeted via ID element retrotransposons. Neuron. 2011; 69:877–884. 21382548 10.1016/j.neuron.2011.02.028 PMC3065018

[97] Tabrez S.S. , Sharma R.D., Jain V., Siddiqui A.A., Mukhopadhyay A. Differential alternative splicing coupled to nonsense-mediated decay of mRNA ensures dietary restriction-induced longevity. Nat. Commun. 2017; 8:306. 28824175 10.1038/s41467-017-00370-5 PMC5563511

[98] Lewis B.P. , Green R.E., Brenner S.E. Evidence for the widespread coupling of alternative splicing and nonsense-mediated mRNA decay in humans. Proc. Natl. Acad. Sci. USA. 2003; 100:189–192. 12502788 10.1073/pnas.0136770100 PMC140922

[99] Chang Y.-F. , Imam J.S., Wilkinson M.F. The nonsense-mediated decay RNA surveillance pathway. Annu. Rev. Biochem. 2007; 76:51–74. 17352659 10.1146/annurev.biochem.76.050106.093909

[100] Hug N. , Longman D., Cáceres J.F. Mechanism and regulation of the nonsense-mediated decay pathway. Nucleic Acids Res. 2016; 44:1483–1495. 26773057 10.1093/nar/gkw010 PMC4770240

[101] Goetz A.E. , Wilkinson M. Stress and the nonsense-mediated RNA decay pathway. Cell. Mol. Life Sci. 2017; 74:3509–3531. 28503708 10.1007/s00018-017-2537-6 PMC5683946

[102] Johnson J.L. , Stoica L., Liu Y., Zhu P.J., Bhattacharya A., Buffington S.A., Huq R., Eissa N.T., Larsson O., Porse B.T. et al. Inhibition of Upf2-Dependent nonsense-mediated decay leads to behavioral and neurophysiological abnormalities by activating the immune response. Neuron. 2019; 104:665–679. 31585809 10.1016/j.neuron.2019.08.027 PMC7312756

[103] Lu J. , Plank T.-D., Su F., Shi X., Liu C., Ji Y., Li S., Huynh A., Shi C., Zhu B. et al. The nonsense-mediated RNA decay pathway is disrupted in inflammatory myofibroblastic tumors. J. Clin. Invest. 2016; 126:3058–3062. 27348585 10.1172/JCI86508 PMC4966300

[104] Ho W.Y. , Agrawal I., Tyan S.-H., Sanford E., Chang W.-T., Lim K., Ong J., Tan B.S.Y., Moe A.A.K., Yu R. et al. Dysfunction in nonsense-mediated decay, protein homeostasis, mitochondrial function, and brain connectivity in ALS-FUS mice with cognitive deficits. Acta Neuropathol. Commun. 2021; 9:9. 33407930 10.1186/s40478-020-01111-4 PMC7789430

[105] Lareau L.F. , Inada M., Green R.E., Wengrod J.C., Brenner S.E. Unproductive splicing of SR genes associated with highly conserved and ultraconserved DNA elements. Nature. 2007; 446:926–929. 17361132 10.1038/nature05676

[106] Song S. , Miranda C.J., Braun L., Meyer K., Frakes A.E., Ferraiuolo L., Likhite S., Bevan A.K., Foust K.D., McConnell M.J. et al. Major histocompatibility complex class I molecules protect motor neurons from astrocyte-induced toxicity in amyotrophic lateral sclerosis. Nat. Med. 2016; 22:397–403. 26928464 10.1038/nm.4052 PMC4823173

