# Supplementary material for: Reactive astrocytes in ALS display diminished intron retention
Source: Nucleic Acids Res. 2021 Mar 4;49(6):3168–84. doi: 10.1093/nar/gkab115 (PMC8034657; doi:10.1093/nar/gkab115)
Supplement: gkab115_Supplemental_Files [file gkab115_supplemental_files.zip › Supplement Figures. Reactive astrocytes in ALS models display dysregulated IR.pdf]

SUPPLEMENTAL FIGURES

Reactive astrocytes in ALS display diminished intron retention

Oliver J. Ziff[1,2,3], Doaa M. Taha[1,2,4]*, Hamish Crerar[1,2]*, Benjamin E. Clarke[1,2], Anob M. Chakrabarti[1,5], Gavin Kelly[1], Jacob Neeves[1,2], Guilia Tyzack[1,2], Nicholas M. Luscombe[1,5,6 †], Rickie Patani[1,2,3 † #]*

Supplementary Figure 1 **3**

Supplementary Figure 2 **5**

Supplementary Figure 3 **7**

Supplementary Figure 4 **9**

Supplementary Figure 5 **11**

Supplementary Figure 6 **13**

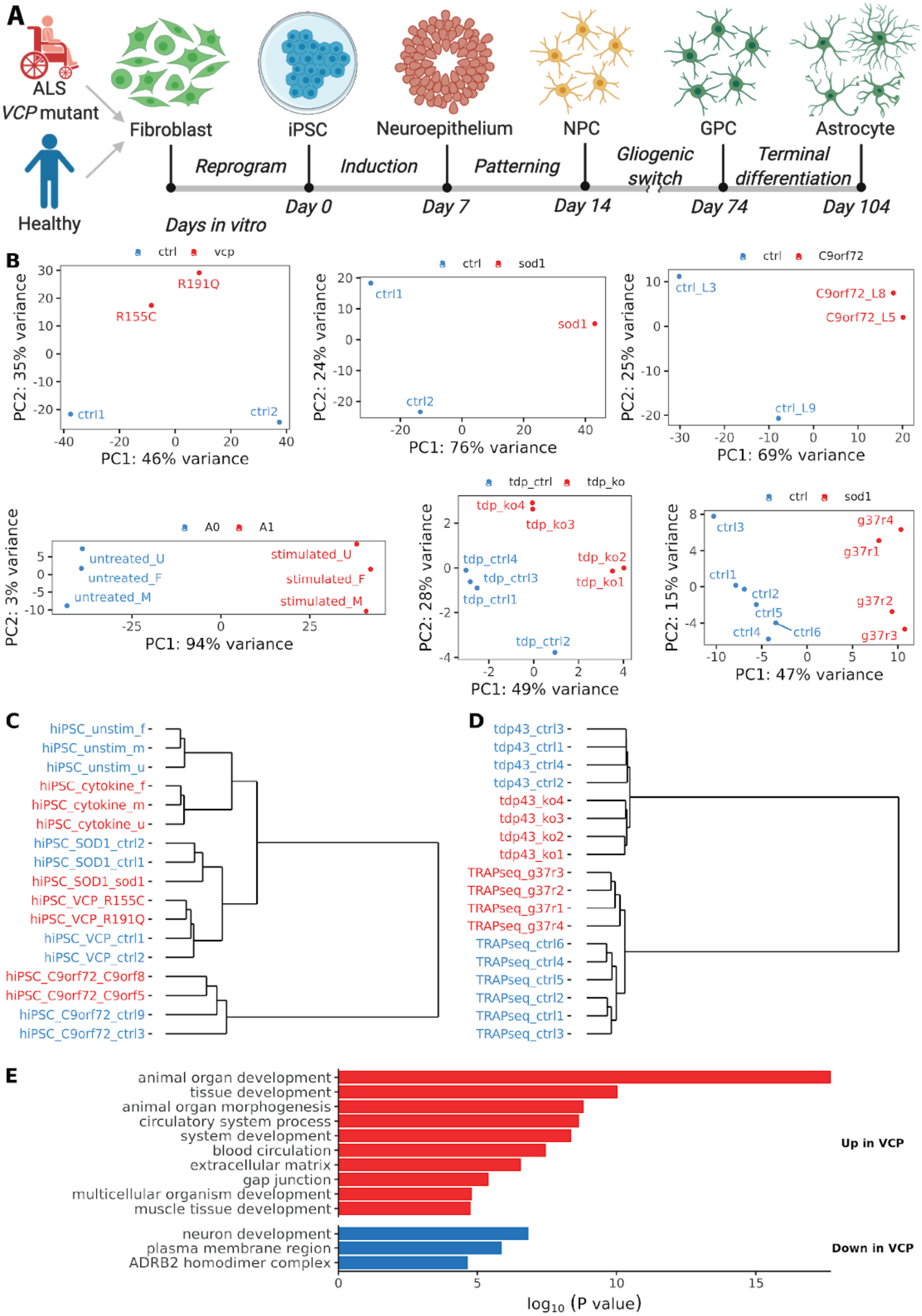
A
ALS
VCP mutant
Healthy
Fibroblast
iPSC
Neuroepithelium
NPC
GPC
Astrocyte
Reprogram
Induction
Patterning
Gliogenic switch
Terminal differentiation
Days in vitro
Day 0
Day 7
Day 14
Day 74
Day 104
B
ctrl
vcp
R191Q
R155C
ctrl1
ctrl2
PC2: 35% variance
PC1: 46% variance
ctrl
sod1
ctrl1
sod1
ctrl2
PC2: 24% variance
PC1: 76% variance
ctrl
C9orf72
ctrl_L3
C9orf72_L8
C9orf72_L5
ctrl_L9
PC2: 25% variance
PC1: 69% variance
A0
A1
untreated_U
untreated_F
untreated_M
stimulated_U
stimulated_F
stimulated_M
PC2: 3% variance
PC1: 94% variance
tdp_ctrl
tdp_ko
tdp_ko4
tdp_ko3
tdp_ctrl4
tdp_ctrl3
tdp_ctrl1
tdp_ko2
tdp_ko1
tdp_ctrl2
PC2: 28% variance
PC1: 49% variance
ctrl
sod1
ctrl3
g37r4
g37r1
ctrl1
ctrl2
ctrl5
ctrl4
ctrl6
g37r2
g37r3
PC2: 15% variance
PC1: 47% variance
C
hiPSC_unstim_f
hiPSC_unstim_m
hiPSC_unstim_u
hiPSC_cytokine_f
hiPSC_cytokine_m
hiPSC_cytokine_u
hiPSC_SOD1_ctrl2
hiPSC_SOD1_ctrl1
hiPSC_SOD1_sod1
hiPSC_VCP_R155C
hiPSC_VCP_R191Q
hiPSC_VCP_ctrl1
hiPSC_VCP_ctrl2
hiPSC_C9orf72_C9orf8
hiPSC_C9orf72_C9orf5
hiPSC_C9orf72_ctrl9
hiPSC_C9orf72_ctrl3
D
tdp43_ctrl3
tdp43_ctrl1
tdp43_ctrl4
tdp43_ctrl2
tdp43_ko4
tdp43_ko3
tdp43_ko2
tdp43_ko1
TRAPseq_g37r3
TRAPseq_g37r2
TRAPseq_g37r1
TRAPseq_g37r4
TRAPseq_ctrl6
TRAPseq_ctrl4
TRAPseq_ctrl5
TRAPseq_ctrl2
TRAPseq_ctrl1
TRAPseq_ctrl3
E
animal organ development
tissue development
animal organ morphogenesis
circulatory system process
system development
blood circulation
extracellular matrix
gap junction
multicellular organism development
muscle tissue development
neuron development
plasma membrane region
ADRB2 homodimer complex
Up in VCP
Down in VCP
0
5
10
15
log10 (P value)

Supplementary Figure 1

A: *iPSC-derived astrocyte differentiation strategy. hiPSC; neural precursor cells (NPC); glial precursor cells (GPC). Numbers represent days in vitro (DIV). The gliogenic switch from NPCs to GPCs takes ~60 DIV as depicted by the timeline break.*
B: *Principal component analyses (PCA) on variance stabilised gene counts across all 6 datasets (VCP mutant hiPSC-derived astrocytes; SOD1 mutant hiPSC-derived astrocytes; C9orf72 mutant hiPSC-derived astrocytes; cytokine stimulated hiPSC-derived astrocytes, in vivo mouse astrocyte TDP43 deletion, and in vivo SOD1 mutant mouse TRAPseq plotted by their coordinates along the first two principal components. Samples are labeled by their cell line and coloured according to mutant/treatment and control groups.*
C-D: *Dendrogram showing unsupervised hierarchical clustering of variance stabilised gene counts across astrocyte datasets. C shows the 4 human datasets and D shows the 2 mouse datasets (separated due to different GTF annotation files used during read alignment).*
E: *Gene ontology terms enriched in up and downregulated differentially expressed genes in VCP vs control astrocytes. For individual genes results see Tables S2.*

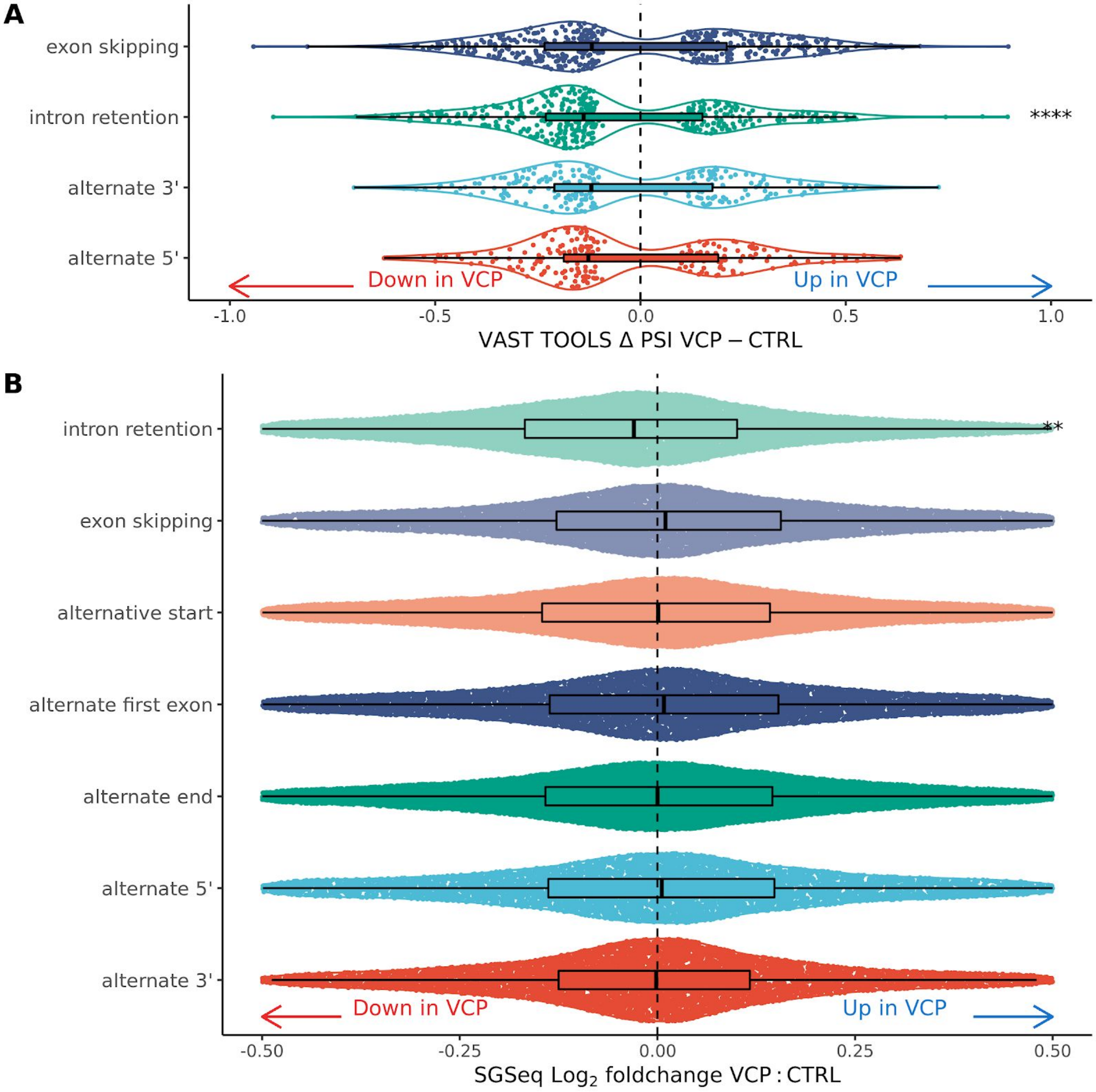

Supplementary Figure 2

A: *Violin plot showing all modes of significant alternative splicing events in VCP versus control astrocytes with VAST-TOOLS (exon skipping n = 464, intron retention n = 406, alternate 3′ n = 221, alternate 5′ n = 200)*

B: *Violin plot of alternative splicing events in VCP versus control astrocytes with SGSeq splice graph tool (intron retention n = 19,576, exon skipping n = 19,215, alternative start n = 38,034, alternate first exon n = 20,118, alternate end n = 40,093, alternate 5′ n = 11,473, alternate 3′ n = 12,397).*

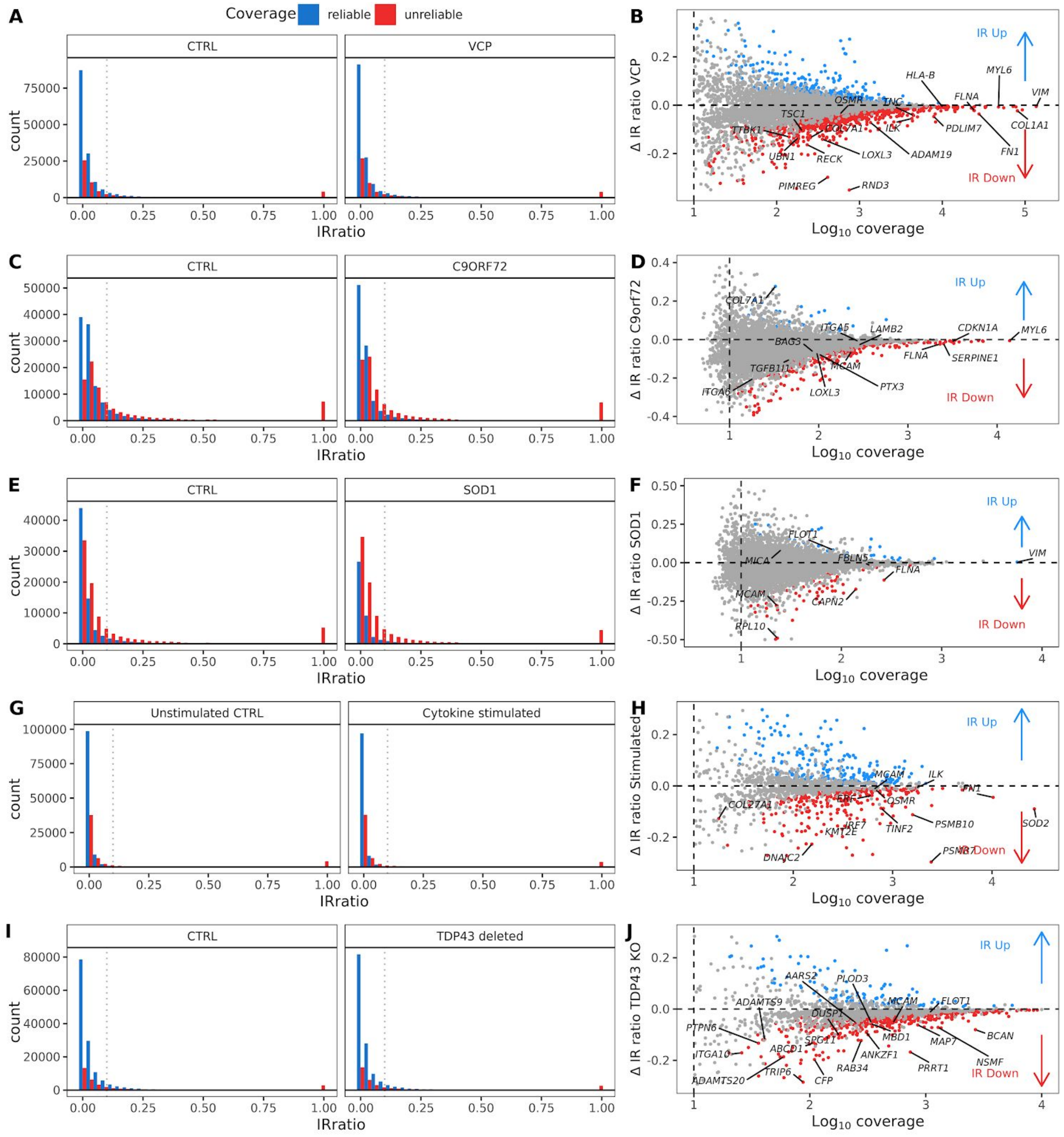

Supplementary Figure 3

Left panels: *Reliable (blue) versus unreliable (red) expressed IR events according to their IR ratio in VCP (A), C9ORF72 (C) , SOD1 (E), cytokine stimulated G) and TDP43 deleted (I) astrocytes. Reliable IR event expression is defined as (1) spliced reads mapping across the 3′ and 5′ flanking exons + intron reads > 10 and (2) > 4 reads from across the 3′ and 5′ flanking exons.*

Right panels: *MA plots of delta IR ratio (mutant minus control astrocytes) plotted against the log10 mean intron and neighbouring exon coverage for VCP (B), C9ORF72 (D) , SOD1 (F), cytokine stimulated (H) and TDP43 deleted (J) astrocytes. Grey points indicate IR events with no significant difference in IR between groups (p < 0.05), red points indicate IR events significantly decreased in mutant and blue indicates IR events significantly increased. Only IR events with log10 mean exonic and intronic coverage > 1 (vertical dotted line) are considered reliable.*

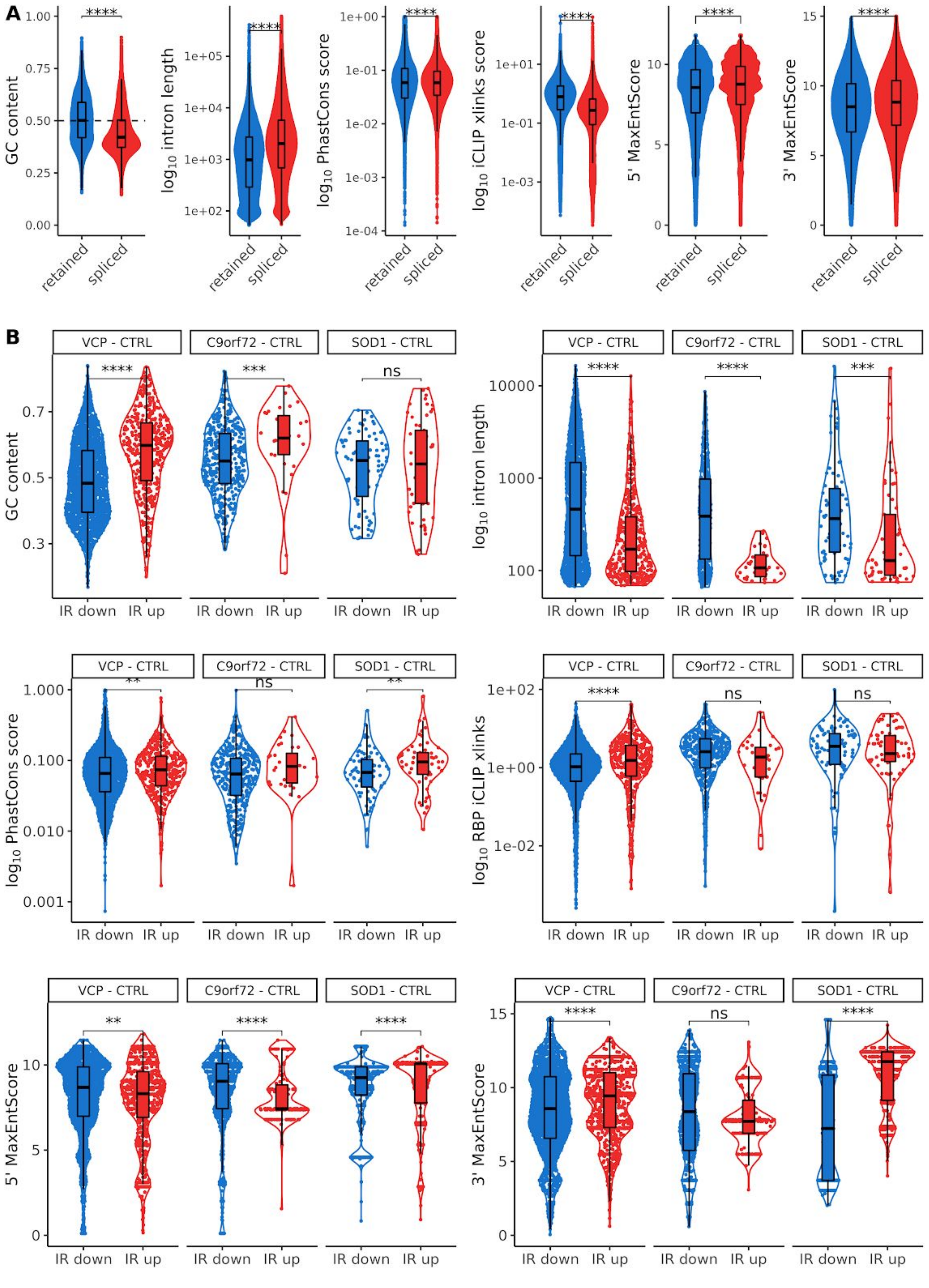
A
GC content
retained
spliced
log10 intron length
log10 PhastCons score
log10 iCLIP xlinks score
5' MaxEntScore
3' MaxEntScore
B
VCP - CTRL
C9orf72 - CTRL
SOD1 - CTRL
IR down
IR up
GC content
log10 intron length
log10 PhastCons score
log10 RBP iCLIP xlinks
5' MaxEntScore
3' MaxEntScore
ns

Supplementary Figure 4

A: *Violin plots showing intron characteristics of retained (n = 28,759) versus non-retained introns (n = 134,185) across all 3 mutant ALS datasets (VCP, C9orf72 and SOD1). Intron characteristics are GC content, intron length, phastCons conservation score, iCLIP crosslink scores with RBPs, Maximum Entropy score from MaxEntScan for 5′ splice sites and 3′ splice sites.*
B: *Intron characteristics in upregulated versus downregulated retained introns in VCP (left), C9orf72 (middle) and SOD1 (right) versus control astrocytes (VCP IR down n = 1,885, VCP IR up n = 424; C9orf72 IR down n = 416, C9orf72 IR up n = 32; SOD1 IR down n = 82, SOD1 IR up n = 57). **** represents adjusted P values from wilcoxon test **** < 0.0001, *** < 0.001, * < 0.05).*

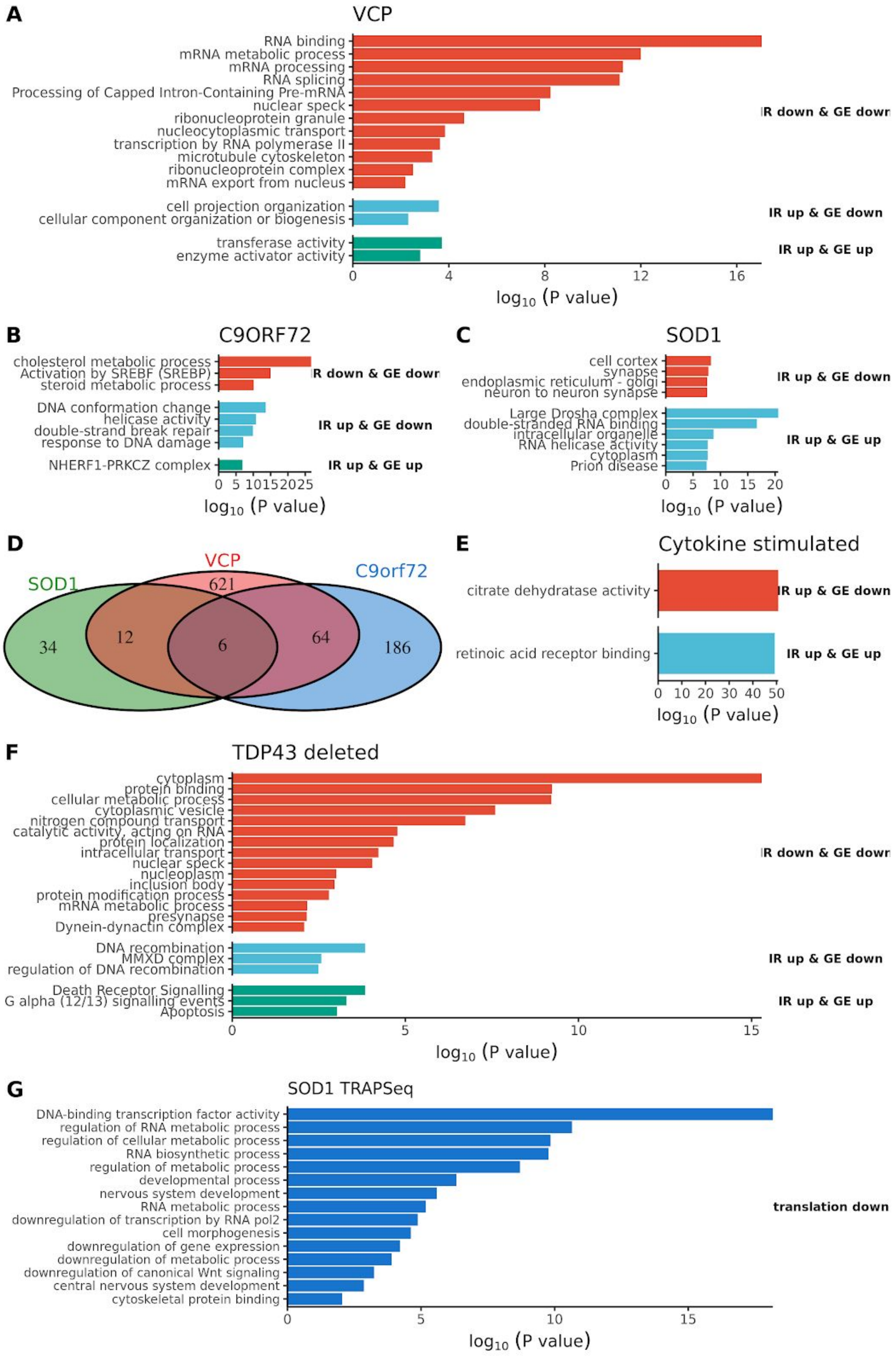
A
VCP
RNA binding
mRNA metabolic process
mRNA processing
RNA splicing
Processing of Capped Intron-Containing Pre-mRNA
nuclear speck
ribonucleoprotein granule
nucleocytoplasmic transport
transcription by RNA polymerase II
microtubule cytoskeleton
ribonucleoprotein complex
mRNA export from nucleus
cell projection organization
cellular component organization or biogenesis
transferase activity
enzyme activator activity
IR down & GE down
IR up & GE down
IR up & GE up
0 4 8 12 16
log10 (P value)
B
C9ORF72
cholesterol metabolic process
Activation by SREBF (SREBP)
steroid metabolic process
DNA conformation change
helicase activity
double-strand break repair
response to DNA damage
NHERF1-PRKCZ complex
IR down & GE down
IR up & GE down
IR up & GE up
0 5 10 15 20 25
log10 (P value)
C
SOD1
cell cortex
synapse
endoplasmic reticulum - golgi
neuron to neuron synapse
Large Drosha complex
double-stranded RNA binding
intracellular organelle
RNA helicase activity
cytoplasm
Prion disease
IR up & GE down
IR up & GE up
0 5 10 15 20
log10 (P value)
D
SOD1
VCP
C9orf72
621
34
12
6
64
186
E
Cytokine stimulated
citrate dehydratase activity
retinoic acid receptor binding
IR up & GE down
IR up & GE up
0 10 20 30 40 50
log10 (P value)
F
TDP43 deleted
cytoplasm
protein binding
cellular metabolic process
cytoplasmic vesicle
nitrogen compound transport
catalytic activity, acting on RNA
protein localization
intracellular transport
nuclear speck
nucleoplasm
inclusion body
protein modification process
mRNA metabolic process
presynapse
Dynein-dynactin complex
DNA recombination
MMXD complex
regulation of DNA recombination
Death Receptor Signalling
G alpha (12/13) signalling events
Apoptosis
IR down & GE down
IR up & GE down
IR up & GE up
0 5 10 15
log10 (P value)
G
SOD1 TRAPSeq
DNA-binding transcription factor activity
regulation of RNA metabolic process
regulation of cellular metabolic process
RNA biosynthetic process
regulation of metabolic process
developmental process
nervous system development
RNA metabolic process
downregulation of transcription by RNA pol2
cell morphogenesis
downregulation of gene expression
downregulation of metabolic process
downregulation of canonical Wnt signaling
central nervous system development
cytoskeletal protein binding
translation down
0 5 10 15
log10 (P value)

Supplementary Figure 5

A-C: *Bar graphs showing curated GO terms for significantly overrepresented functional categories (FDR < 0.05) determined by gene ontology analysis of genes from the other 3 IR and gene expression (GE) categories not shown in Figs. 2E-G in VCP, C9ORF72 and SOD1. See Table S5 for overlapping genes between datasets in IR and GE direction.*
D: *Venn diagram showing the number of overlapping genes with decreased intron retention and increased gene expression in VCP (red), C9orf72 (blue) and SOD1 (green).*
E-F: *Bar graphs showing curated GO terms for significantly overrepresented functional categories (FDR < 0.05) determined by gene ontology analysis of genes from the other 3 IR and gene expression (GE) categories not shown in Figs. 3H-I in cytokine stimulated and TDP43 deleted.*
G: *Bar graph showing curated significantly overrepresented functional categories (FDR < 0.05) determined by gene ontology analysis of genes with decreased translation in* $SOD1^{G37R}$ *vs control mouse spinal cord astrocytes. See Fig. 3J for genes increased in translation.*

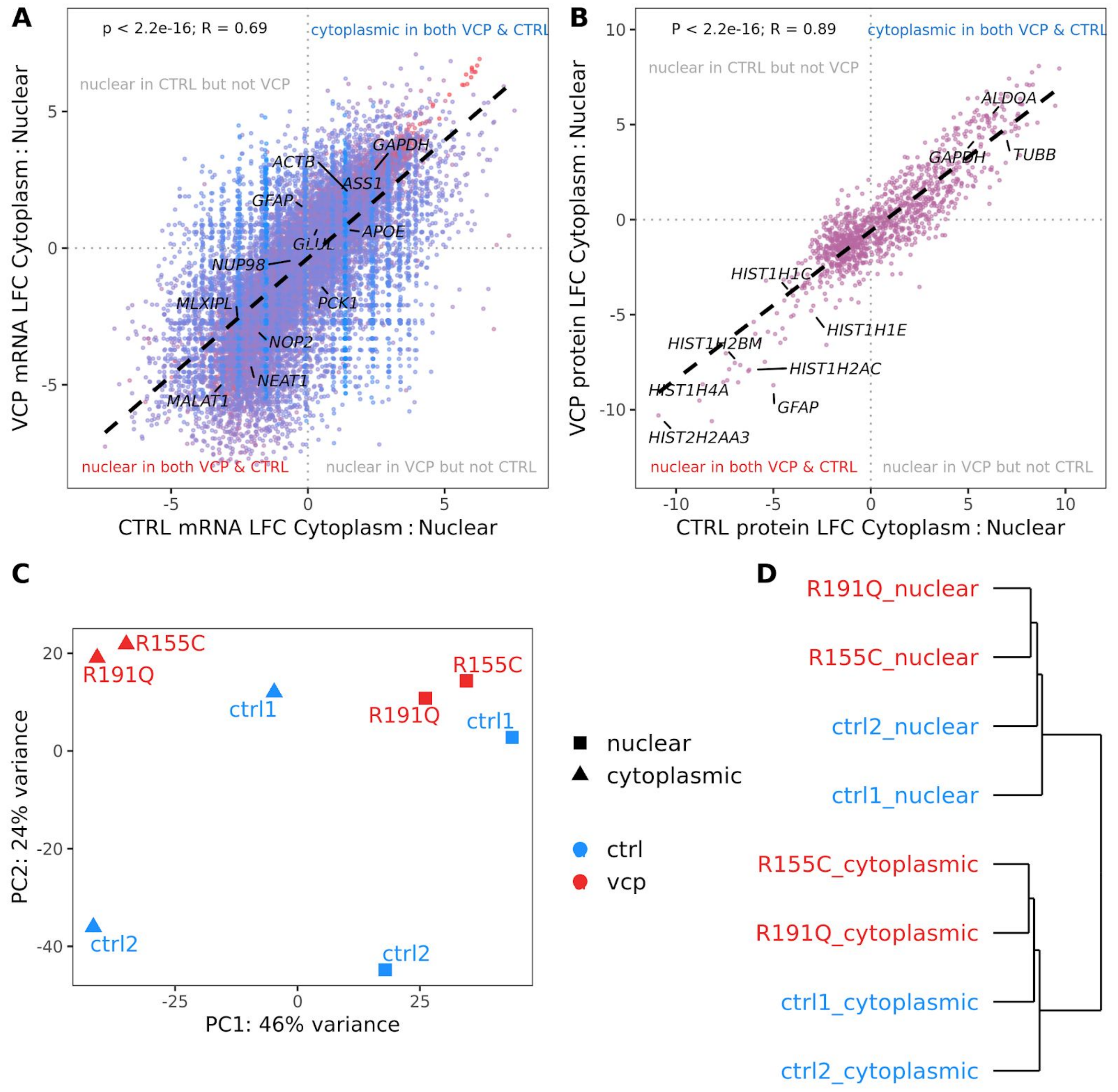

Supplementary Figure 6

A-B: *Scatterplot showing quality control of fractionation using RNAseq (A) and mass spectrometry (B). Log2 fold change (LFC) in gene expression (GE) and protein expression in cytoplasmic versus nuclear fractions in control (x-axis) against VCP (y-axis). RNAseq dots coloured by log_{10} mean expression (blue = low, red = high). Grey dotted lines indicate the points of no difference between cytoplasm and nuclear fraction expression. Black dashed black line represents the linear regression line (RNAseq R = +0.69; mass spectrometry R = +0.89). Labels represent nuclear and cytoplasmic gene markers as reported in (Bahar Halpern et al. 2015).*

C: *Principal component analysis (PCA) on normalised gene counts across astrocyte nuclear and cytoplasmic fractions. Samples are plotted by their coordinates along PC1 (46% of variance) and PC2 (24% of variance). Nuclear astrocyte fractions shown as triangles and cytoplasmic fractions as circles. VCP samples are red and control samples are blue.*

D: *Dendrogram showing unsupervised hierarchical clustering of RNAseq variance stabilised gene counts across astrocyte fractionated samples.*

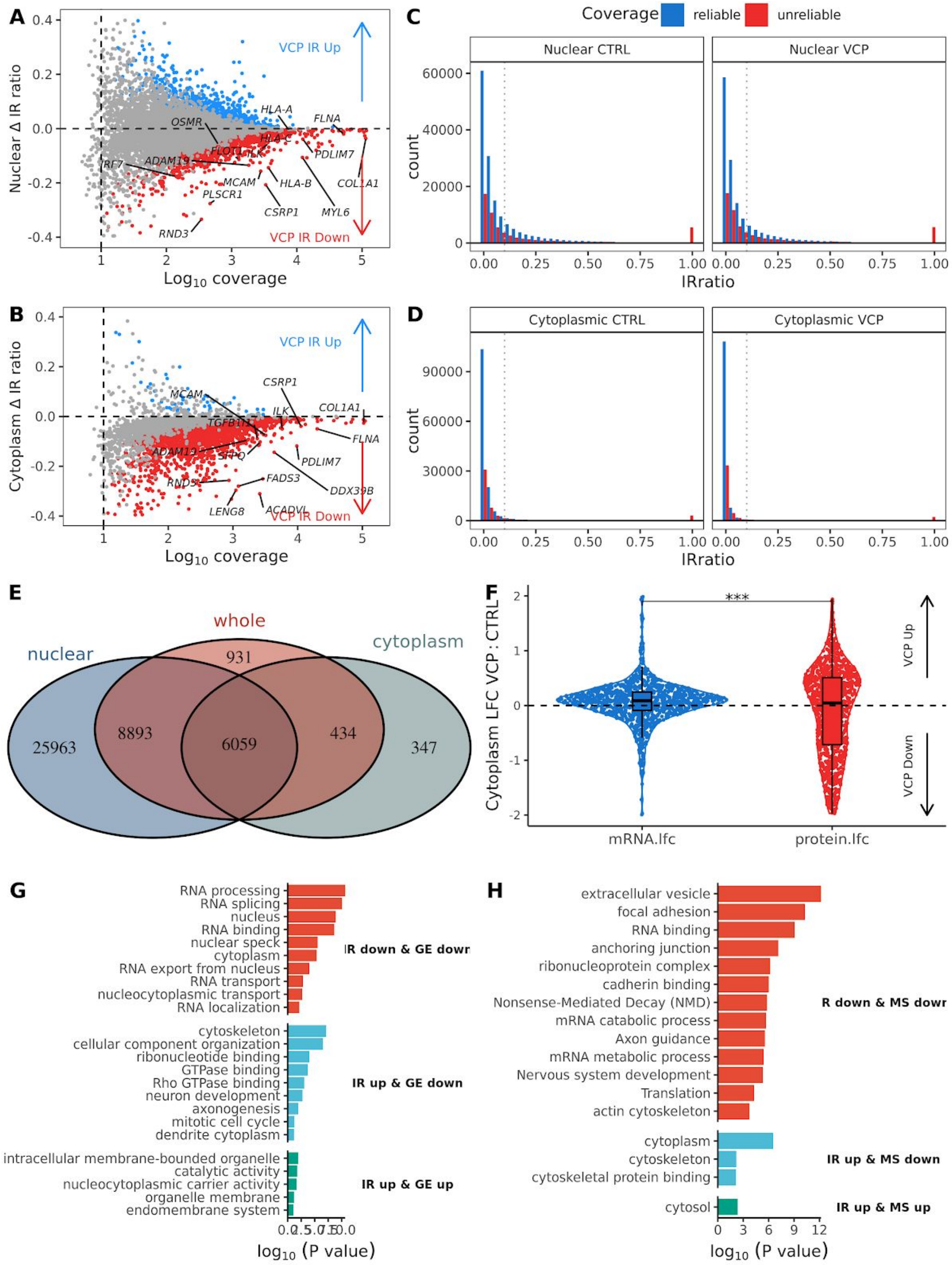

Supplementary Figure 7

A-B: *MA plot of Δ IR ratio (VCP minus control) in the nuclear (A) and cytoplasmic (B) fractions plotted against the log10 intron event coverage. Grey points indicate IR events with no significant difference in IR*

between VCP and control (with FDR < 0.05), red indicates IR events significantly decreased in VCP and blue indicates IR events significantly increased. Only IR events with log10 mean exonic and intronic coverage > 1 (vertical dotted line) were considered reliably expressed.
C-D: *Reliable (blue) versus unreliable (red) expressed IR events according to their difference in IR ratio in nuclear (C) and cytoplasmic fractions (D). Reliable IR event expression is defined as (1) the number of spliced reads mapping across the 3′ and 5′ flanking exons + intron reads > 10 and with (2) > 4 reads from across the 3′ and 5′ flanking exons.*
E: *Venn diagram of the numbers of unique retained introns in either VCP or control conditions, comparing whole astrocytes (red) with nuclear (blue) and cytoplasmic (green) fractions.*
F: *Violin plot showing variance scaled log fold changes (LFC) in VCP vs CTRL (y-axis) for cytoplasmic gene expression (mRNA) and cytoplasmic mass spectrometry (protein; n = 578) amongst genes with detected cytoplasmic protein. Protein LFCs were significantly lower than mRNA LFCs (wilcoxon test p = 0.0004).*
G: *GO categories associated with nuclear IR and cytoplasmic gene expression (GE) for 3 groups not shown in Fig. 4I.*
H: *GO categories associated with nuclear IR and cytoplasmic mass spectrometry (MS) for 3 groups not shown in Fig. 4M.*

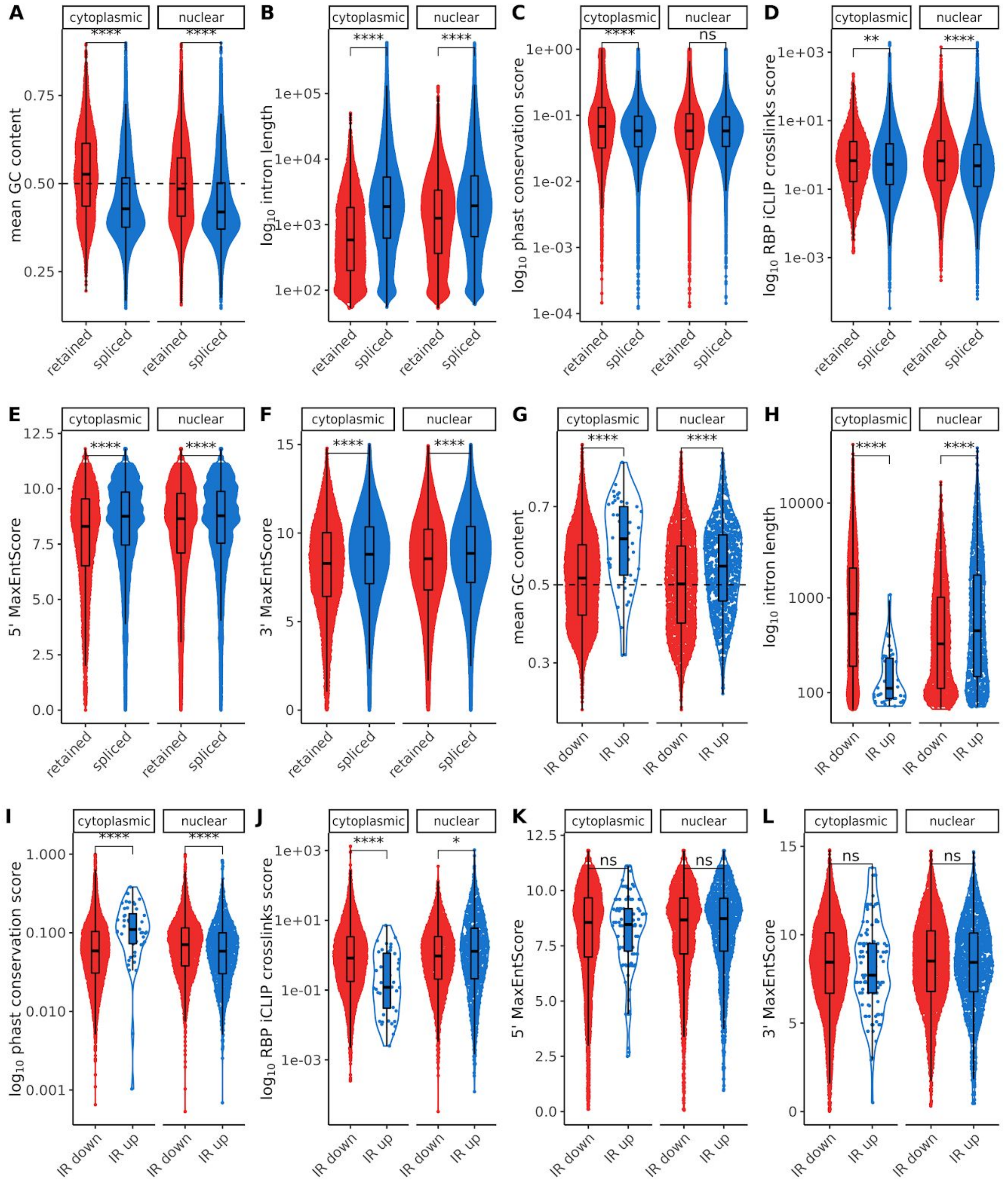

Supplementary Figure 8

A-F: *Violin plots showing intron characteristics (A, intron length; B, GC content; C, phastCons conservation score; D, iCLIP crosslinking with RBPs; E, Maximum entropy score of 5′ splice site; and F, Maximum entropy of 3′ splice site) of retained versus non-retained introns (left, nuclear retained n = 41,138, nuclear spliced n = 119,203; cytoplasmic retained n = 7,063, cytoplasmic spliced n = 154,151)*

G-L:** Intron characteristics of upregulated versus downregulated retained introns in VCP compared with control astrocytes (right, nuclear IR down n = 1,882, nuclear IR up n = 1,033; cytoplasmic IR down n = 5,089, cytoplasmic IR up n = 49). Results for cytoplasmic fractions are shown on the left facet and nuclear fractions on the right facet of each figure. * represents adjusted P values from t-test < 0.0001, *** < 0.001, * < 0.05).*

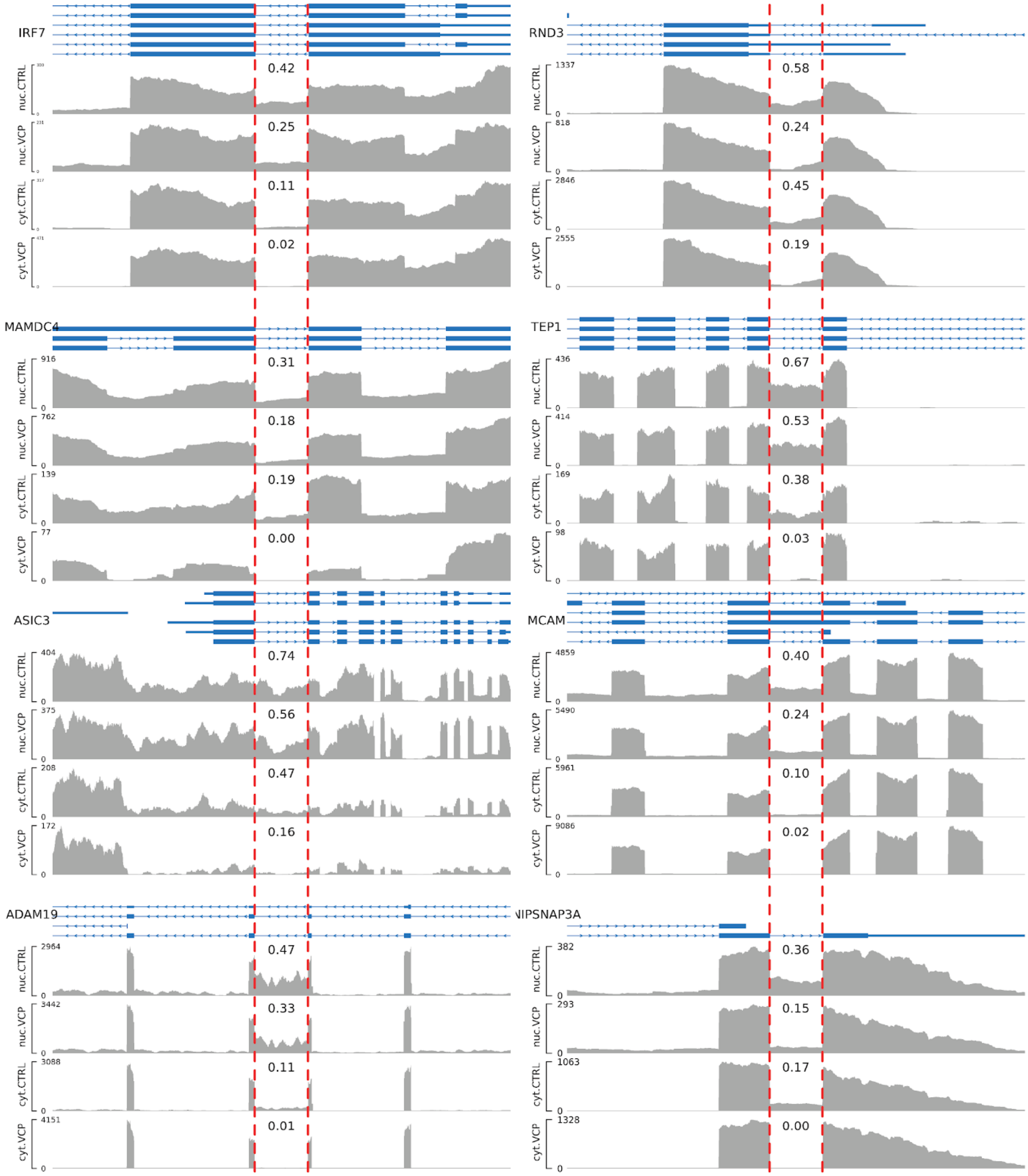

Supplementary Figure 9

Coverage plots of reads per million from mRNA sequencing shown for IRF7, RND3, MAMDC4, TEP1, ASIC3, MCAM, ADAM19 and NIPSNAP3A genes where intron retaining transcripts are lost from the nucleus and cytoplasm in VCP vs control. Alignment tracks are for nuclear control (top), nuclear VCP (second), cytoplasmic control (third), and cytoplasmic VCP (bottom). Gene annotation is shown above the alignment tracks with exons (thick blue boxes), UTRs (thinner blue boxes) and introns (horizontal blue arrowed line). Vertical red dashed lines show the retained intron and the IR ratio value is labelled.
